# A mechanistic model of cross-bridge migration in RBC aggregation and disaggregation

**DOI:** 10.3389/fbioe.2022.1049878

**Published:** 2022-12-06

**Authors:** Swe Soe Maung Ye, Sangho Kim

**Affiliations:** Department of Biomedical Engineering, National University of Singapore, Singapore, Singapore

**Keywords:** red blood cell (RBC), fibrinogen bridging, RBC aggregation, RBC disaggregation, RBC doublet, bridging theory, cross-bridge migration, cell adhesion

## Abstract

Red blood cells (RBCs) clump together under low flow conditions in a process called RBC aggregation, which can alter RBC perfusion in a microvascular network. As elevated RBC aggregation is commonly associated with cardiovascular and inflammatory diseases, a better understanding of aggregation is essential. Unlike RBC aggregation in polymer solutions which can be well explained by polymer depletion theory, plasma-mediated RBC aggregation has features that best match explanations with cross-bridging mechanisms. Previous studies have demonstrated the dominant role of fibrinogen (Fg) in promoting aggregate formation and recent cell-force spectroscopy (CFS) experiments on interacting RBC doublets in plasma have reported an inverse relationship between disaggregation force and the adhesive contact area between RBCs. This has led investigators to revisit the hypothesis of inter-RBC cross-bridging which involves cross-bridge migration under interfacial tension during the forced disaggregation of RBC aggregates. In this study, we developed the cross-bridge migration model (CBMM) in plasma that mechanistically represents the migrating cross-bridge hypothesis. Transport of mobile Fg cross-bridges (mFg) was calculated using a convection-diffusion transport equation with our novel introduction of convective cross-bridge drift that arises due to intercellular friction. By parametrically transforming the diffusivity of mFg in the CBMM, we were able to match experimental observations of both RBC doublet formation kinematics and RBC doublet disaggregation forces under optical tweezers tension. We found that non-specific cross-bridging promotes spontaneous growth of adhesion area between RBC doublets whereas specific cross-bridging tends to prevent adhesion area growth. Our CBMM was also able to correlate Fg concentration shifts from healthy population blood plasma to SLE (lupus) condition blood plasma with the observed increase in doublet disaggregation forces for the RBC doublets in SLE plasma.

## Introduction

An important feature of red blood cells (RBCs) in micro-hemorheology is their tendency to clump into tightly packed clusters under low flow conditions. This reversible adhesion state where cluster formation is balanced by shear-induced dispersion between RBCs is defined as RBC aggregation (Fåhraeus, 1929; Schmid-Schonbein et al., 1968; Somer and Meiselman, 1993). Through collective resistance of aggregate clusters against flow, increased hematocrit partitioning asymmetry at pre-capillary bifurcations (Yin et al., 2013) can reduce effective perfusion of RBCs in capillary and venular networks (Kim et al., 2006; Namgung et al., 2015). Furthermore, RBC aggregation may promote fluid extravasation in capillaries (Knisely et al., 1947) by increasing blood viscosity and pressure in post-capillary regions (Somer and Meiselman, 1993; Johnson et al., 1999; Fedosov et al., 2011b). In pathology, elevated RBC aggregation (hyper-aggregation) is a commonly reported condition in patients suffering from cardiovascular disease (Lee et al., 2007; Arbel et al., 2012), inflammatory disease (Ami et al., 2001), diabetes (van Haeringen et al., 1973; Schmid-Schonbein and Volger, 1976) and hypertension (Lominadze et al., 1998). Hyper-aggregation is also a predictor for fatal complications in the post-surgery management of myocardial infarction (Sargento et al., 2005). As such, the pathophysiological impact of RBC aggregation is significant. Clinical interventions for arresting hyper-aggregation and its compounding effect on circulatory disorders may benefit from a deeper understanding of the fundamental mechanisms separating benign aggregation from hyper-aggregation.

RBC aggregation is mediated by hydrodynamic factors such as shear stress which inhibit stable aggregate formation (Schmid-Schonbein et al., 1968; Ami et al., 2001) and the local hematocrit for which aggregation rate has been found to occur optimally at physiological hematocrits (Deng et al., 1994). Plasma and suspension factors also influence aggregation through cross-bridging or depletion interactions arising from macromolecules in the suspending medium and their interactions with the RBC surface (Ami et al., 2001; Neu and Meiselman, 2002; Lee et al., 2016b). In this regard, one trend in the development of mechanistic models of RBC aggregation has been focused on depletion theory. Depletion theory for RBC aggregation suggests that a polymer chemical potential (osmotic pressure gradient) is established between the polymer-poor depletion zone on the RBC surface and the surrounding polymer-rich bulk solution. The resulting expulsion of water from the intercellular gap gives rise to an attractive force between cells which is balanced against the intercellular electrostatic repulsion at nanometer separation distances (Neu and Meiselman, 2002). The depletion model for RBC aggregation has been extensively developed to include effects of the glycocalyx structure (Rad et al., 2014), solution isotonicity and RBC aging (Neu et al., 2003), and polymer molecular weight (Rad et al., 2009). Consequently, the depletion model for RBC aggregation forms the basis of RBC aggregation induction in microrheological experiments using polymer simulants like dextran in phosphate buffered saline (PBS) solution.

Unlike polymer simulants however, RBC aggregation in blood plasma may not be dominated by depletion mechanics. Indeed, one study has highlighted that while RBC aggregation readily occurs between two RBCs in blood plasma, RBCs in PBS suspensions containing only physiological concentrations of Fibrinogen (Fg) and Albumin (Alb) do not form stable aggregates (Lee et al., 2016b). Another study examining the erythrocyte sedimentation rate (ESR) of RBC populations in various permutations of blood plasma constituents concluded that cooperativity between Fg, Alb and immunoglobins in plasma was required for physiological levels of aggregation to occur. Plasma with Alb alone or in binary solution with dextran actually inhibited aggregation (Reinhart and Nagy, 1995). Hence these phenomena in plasma variants cannot be explained by depletion theory alone.

Contrary to the effort in developing depletion models of RBC aggregation, researchers for cross-bridging theory have instead focused on experimental verification of possible cross-bridging mechanisms and forces. Supporting specific cross-bridging are observations that Fg can specifically bind with receptors on the RBC (Lominadze and Dean, 2002; De Oliveira et al., 2012; Sokolova et al., 2014) and also specifically bind to other Fg with bond strengths up to 10 pN (Litvinov et al., 2007). Conversely many studies support non-specific bridging interactions to be primary coordinators of RBC aggregation in plasma. Firstly, Fg receptors are too sparsely distributed to be primary coordinators of physiological aggregation (Lominadze and Dean, 2002). Secondly, Fg to RBC specific bonds (FgR) are 20–80 pN strong (Carvalho et al., 2010; Carvalho et al., 2011) and this exceeds the ∼30 pN disaggregation forces measured in plasma-suspended RBC aggregates (Khokhlova et al., 2012; Lee et al., 2016b). While these findings give piecemeal insight into Fg physiochemistry on the RBC surface, there has been no coordinated effort to update the cross-bridge model of RBC aggregation based on a holistic debate about these findings.

Recently, cell force spectroscopy (CFS) techniques have developed a deeper mechanistic understanding of doublet-level aggregation through measurement of the forces of disaggregation under varying stress application scenarios and suspending media constituents (Bronkhorst et al., 1997; Khokhlova et al., 2012; Steffen et al., 2013; Lee et al., 2016a; Lee et al., 2016b; Lee et al., 2017). In addition to quantifying the dominant contribution of Fg in promoting RBC aggregation (Lee et al., 2016a), CFS studies on RBC doublet disaggregation have reported an increase in adhesive force between RBCs despite the gradual loss of contact area when a doublet is forcibly sheared apart (Khokhlova et al., 2012; Lee et al., 2016b). This phenomenon cannot be explained by depletion theory or existing models of cross-bridge induced RBC aggregation. The authors of (Lee et al., 2016a) have hypothesized that RBC doublet disaggregation in plasma may pull cross-bridge tethers towards the doublet contact regions *via* membrane elastic tension, similar to a T-cell and target cell adhesion scenario (Tozeren et al., 1989). Instead, in this work, we propose that intercellular friction causes weakly interacting mobile Fg bridges on RBC surfaces to drift in the direction of intercellular friction. Accordingly, we have developed a tunable cross-bridge migration model (CBMM) of RBC aggregation in plasma that mechanistically elucidates the necessary conditions and assumptions for the migrating cross-bridge hypothesis. This model was tuned to directly match 1) aggregate formation kinematics in and doublet formation experiments and 2) doublet disaggregation forces measured in CFS experiments.

## Materials and methods

To study the interfacial forces mediating inter-RBC adhesion in plasma-mediated RBC aggregation, we performed numerical simulations of a two-cell RBC doublet system under doublet formation and forced disaggregation scenarios. The construction of the numerical model entails the RBC deformation model with the coarse-grained particle model of the RBC membrane, the adhesion potential and intercellular friction model for inter-RBC interaction and development of the cross-bridge migration model for adhesion potential modulation in the interface dynamics.

### Adhesion force schemes for specific and non-specific cross-bridges

**FIGURE 1:**
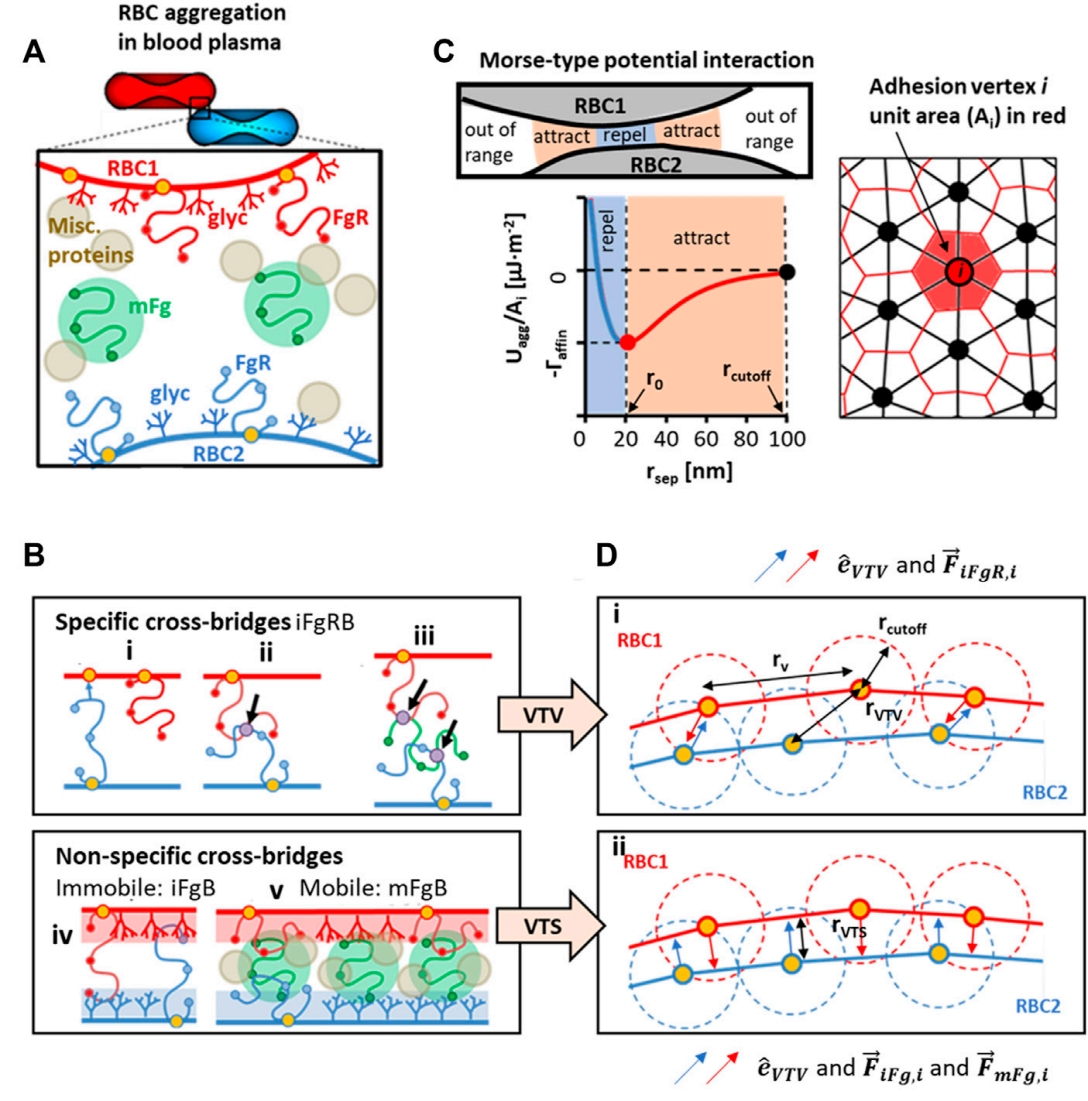
Conceptualization and modeled representation of cross-bridging possibilities in RBC aggregation. **(A)** Contributors to plasma-mediated RBC aggregation is likely to require cooperativity between receptor-bound Fg (FgR), surface adsorbed Fg (mFg) and other plasma proteins and factors. **(B)** Schematics of possible Fg to RBC surface interactions that can categorically be summarized into specific cross-bridges (iFgRB) and non-specific cross-bridges that are immobile (iFgB) and mobile (mFgB). **(C)** The Morse-type potential model used for calculating interaction energy at adhesion vertices and the median-dual area element around vertex i used to calculate local adhesion area **(D)** i)The specific interaction scenarios in Ci, Cii , Ciii are represented by the vertex-to-vertex (VTV) scheme. ii) The non-specific bridging scenarios in Civ and Cv are represented by the vertex-to-surface (VTS) scheme for distance and force calculation.

We have focused our attention on Fg as the key aggregation-inducing protein due to its dominant role in mediating the speed and mechanism of RBC aggregate formation in blood plasma (Maeda et al., 1987; Lee et al., 2016a). Collating the literature on possible Fg-interactions in the plasma scenario, we summarized three types of possible Fg-based cross bridges in Figure 1A,B. The first, iFgRB is a specific cross-bridging generalization that encompasses three subtypes: FgR_2_ cross-bridge, where Fg is specifically bound to two receptors from pairing RBCs (Figure 1Bi); FgR-FgR cross-bridge where two receptor bound Fg (FgR) from pairing RBCs bind to one another at specific Fg domains (Figure 1Bii); and the FgR-Fg-FgR cross-bridge is a specific binding between non-receptor-bound Fg and two FgR to form a multi-Fg cross-bridging complex (Figure 1Biii). The immobile non-specific iFgB is a cross-bridge which involves non-specific and weak interactions between FgR and Fg-associating glycocalyx surface groups (glyc) on the neighboring RBC surface (Figure 1Biv). The mFgB cross-bridges are weak mobile cross-bridges (FgR-mFg-FgR, glyc-mFg-glyc, FgR-mFg-glyc) in the form of mFg weakly associating with FgR and glyc (Figure 1Bv). Please note that in our consideration, only mFgB cross-bridges are mobile.

For the aggregation model construction, we begin with the Morse-type potential model (MP) (Liu and Liu, 2006):

$$U_{agg,i}=\left\{\begin{array}{c} \Gamma_{affin}\left[e^{2\beta \left(r_{0}-r_{sep}\right)}-2e^{\beta \left(r_{0}-r_{sep}\right)}\right]A_{i}\;if\;r_{sep}\leq r_{cutoff}\;\\ 0\;if\;r_{sep}>r_{cutoff} \end{array}\right.$$ (1)

where $U_{agg,i}$ is the interaction energy, i is the mesh vertex at which the calculation is performed, $\Gamma_{affin}$ is the adhesion affinity between the RBC surfaces arising from cross-bridging; β is the interaction spatial decay constant; $r_{sep}$ is the separation distance between interacting regions of the two RBCs; $r_{0}$ is the zero-force separation distance at which inter-surface attraction is negated by the electro-static repulsion forces arising from the overlapping electric double layer between RBCs; $r_{cutoff}$ is the cut-off separation distance beyond which interaction forces vanish; and $A_{i}$ is the surface area of the median-dual control volume around vertex i (see Figure 1C).

We recast the MP (Eq. 1) for mechanistic representation of three general categories of cross-bridging in the cross-bridge migration model (CBMM). Affinity contribution from iFgRB immobile specific cross-bridges is given by $\Gamma_{iFgR}$ . Affinity contribution from iFgB immobile non-specific cross-bridges is given by $\Gamma_{iFg}$ . Affinity contribution from mFgB mobile non-specific cross-bridges is given by $\Gamma_{mFg}$ . The total affinity is thus given:

$$\Gamma_{affin}=\Gamma_{iFgR}+\Gamma_{iFg}+N_{mFg}∙\Gamma_{mFg}\;;N_{mFG}=N_{mFg\;RBC1}+N_{mFg\;RBC2}$$ (2)

where $N_{mFg\;RBC}$ is the relative density of mFg adsorbed on the RBC surface and subscripted suffixes “1” and “2” indicate RBC1 and RBC2 in the doublet–note that total mFg density ( $N_{mFg}$ ) in the intercellular gap is given by the sum of $N_{mFg\;RBC}$ from both RBC surfaces. If we consider the absence of mFg, then the CBMM reduces to the uniform affinity model (UAM) where $\Gamma_{affin}=\Gamma_{iFgR}+\Gamma_{iFg}$ (only iFgRB and iFgB cross-bridges).

Forces of aggregation arising from the three different cross-bridges are as follows:

$${\vec{F}}_{iFgR,i}=\left\{\begin{array}{c} \Gamma_{iFgR}A_{i}\left[2{\beta e}^{\beta \left(r_{0}-r_{VTV}\right)}-{2\beta e}^{2\beta \left(r_{0}-r_{VTV}\right)}\right]{\hat{e}}_{VTV}\;if\;r_{VTV}\leq r_{cutoff}\;\\ 0\;if\;r_{VTV}>r_{cutoff} \end{array}\right.$$ (3a)

$${\vec{F}}_{iFg,i}=\left\{\begin{array}{c} \Gamma_{iFg}A_{i}\left[2{\beta e}^{\beta \left(r_{0}-r_{VTS}\right)}-{2\beta e}^{2\beta \left(r_{0}-r_{VTS}\right)}\right]{\hat{e}}_{VTS}\;if\;r_{VTS}\leq r_{cutoff}\;\\ 0\;if\;r_{VTS}>r_{cutoff} \end{array}\right.$$ (3b)

$${\vec{F}}_{mFg,i}=\left\{\begin{array}{c} N_{mFg}∙\Gamma_{mFg}A_{i}\left[2{\beta e}^{\beta \left(r_{0}-r_{VTS}\right)}-{2\beta e}^{2\beta \left(r_{0}-r_{VTS}\right)}\right]{\hat{e}}_{VTS}\;if\;r_{VTS}\leq r_{cutoff}\;\\ 0\;if\;r_{VTS}>r_{cutoff} \end{array}\right.$$ (3c)

$${\vec{F}}_{agg,i}={\vec{F}}_{iFgR,i}+{\vec{F}}_{iFg,i}+{\vec{F}}_{mFg,i}$$ (3d)

Note that we see the different application of direction vectors ${\hat{e}}_{VTV}$ *versus* ${\hat{e}}_{VTS}$ and distance scalar $r_{VTV}$ and $r_{VTS}$ between cross-bridge types. Specific cross-bridges are represented by the vertex to vertex scheme (VTV) where the ${\vec{F}}_{iFgR,i}$ is applied along ${\hat{e}}_{VTS}$ which is the unit direction vector from vertex i to the neighboring RBC adhesion vertex (Figure 1Di). Conversely, in the vertex to surface (VTS) scheme ${\vec{F}}_{iFg,i}$ , ${\vec{F}}_{iFg,i}$ and $r_{VTS}$ are evaluated using the normal distance vector ( ${\hat{e}}_{VTS}$ ) between the mesh adhesion vertices and the nearest neighboring triangular surface elements on the pairing RBC surface (Figure 1Dii).

### Mobile cross-bridge transport in the cross-bridge migration model

**FIGURE 2:**
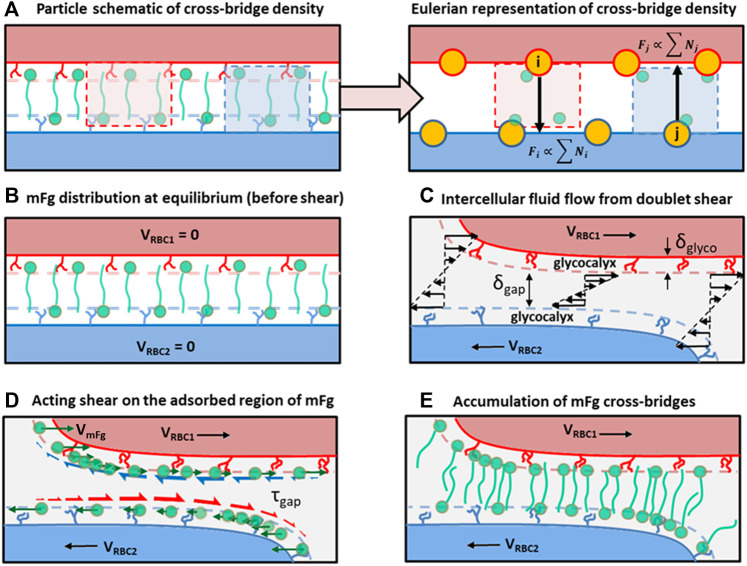
Schematics of the cross-bridge migration model (CBMM) and its conceptual development. **(A)** A particle to Eulerian depiction of the cross-bridge density represented at the adhesion vertices. **(B)** Homogenous distribution of cross-bridges at the adhesion interface at doublet formation equilibrium, which represents the initial condition in our doublet formation/disaggregation simulations. **(C)** Intercellular Couette flow that develops due to the applied displacement on RBCs in the doublet. **(D)** Shear stress acting on adsorbed regions of mFg causing relative velocity drift between mFg and the RBC surface adsorbing mFg. Green arrows represent the mFg velocity that deviates from RBC velocity due to friction. Red half arrows represent the frictional drag force acting on mFg adsorbed on RBC2 surface. Blue half arrows represent the frictional drag force acting on mFg adsorbed on RBC1 surface. **(E)** The resulting cross-bridge accumulation in the intercellular adhesion region from weak association between the non-adsorbed mFg tails and pairing RBC surfaces.

The distribution of $N_{mFg\;RBC}$ in the CBMM is updated by the Eulerian implementation of the planar convection-diffusion transport equation:

$$\frac{dN_{mFg\;RBC}}{dt}=D_{mFg}\nabla^{2}N_{mFg\;RBC}-\vec{\nabla }∙{\vec{V}}_{drift}N_{mFg\;RBC}+{\dot{S}}_{adsorp}$$ (4)

where $N_{mFg\;RBC}$ is a normalized concentration set to 1 across the cell surface at the initialization of simulations to represent the uniform distribution of mFg *via* diffusion for RBC doublets that have been allowed to form over a long time (Figure 2B). $D_{mFg\;}$ is the surface diffusivity of the surface-adsorbed mFg, ${\dot{S}}_{adsorp}$ is the surface source-sink term to account for the net flux of mFg diffusion onto the RBC surface layer due to bulk concentration gradients and the equilibrium adsorption balance. The second right-hand side term in Eq. 4 is the convective transport term where ${\vec{V}}_{drift}$ is the drift velocity of the adsorbed mFg relative to the adsorption surface velocity which we represent in the CBMM as a linear function of the intercellular surface separation distance ( $\delta_{gap}$ ):

$${\vec{V}}_{drift}=\left\{\begin{array}{c} 0.5\left({\vec{V}}_{RBC\;neighbor}-{\vec{V}}_{RBC}\right)\;\frac{\delta_{Fg}{-\delta }_{gap}}{\delta_{Fg}}\;for\;\delta_{gap}<\delta_{Fg}\\ 0\;for\;\delta_{gap}>\delta_{Fg} \end{array}\right.$$ (5)

where ${\vec{V}}_{RBC\;neighbor}$ and ${\vec{V}}_{RBC}$ are the velocities of the adjacent neighboring surface and adsorption surface respectively. $\delta_{gap}=r_{sep}-{2r}_{glyco}$ where $r_{glyco}$ is the glycocalyx height on the RBC surface (Figure 2C) and $\delta_{Fg}$ is the hydrodynamic radius of the surface-adsorbed mFg (∼45 nm based on aqueous Fg (Kollman et al., 2009; Zuev et al., 2017)). The mathematical expression assumes that mFg on the RBC surfaces away from the intercellular space ( $\delta_{gap}=\infty$ ) are not subjected to high intercellular friction ( $\tau_{gap}$ —schematically represented by the half arrows in Figure 2D) and will follow the velocity of the adsorption surface. Furthermore, intercellular gaps larger than the hydrodynamic radius ( $\delta_{gap}>\delta_{Fg}$ ) of the bridging mFg particle are assumed to allow mFg to effectively follow the surface velocity of the moving membrane since the intercellular friction is weak in these regions (Figures 2C,D). For regions with intercellular gaps narrower than the hydrodynamic radius of the mFg, the adsorbed region of mFg will be subjected to strong $\tau_{gap}$ and will experience a drift velocity with respect to its adsorption surface. The drift velocity is our phenomenological representation of adsorbed regions of mFg sliding along the RBC surface to new locations to accumulate cross-bridges locally (Figure 2E).

We also considered the effect of normal fluxes contributing to surface concentration of adsorbed Fg through the ${\dot{S}}_{adsorp}$ term in Eq. 4. The expression for representing ${\dot{S}}_{adsorp}$ in the CBMM is as follows:

$${\dot{S}}_{adsorp}=\left\{\begin{array}{c} J_{constant}+J_{diffus}\;if\;r_{sep}\geq \delta_{Fg}\\ 0\;if\;r_{sep}<\delta_{Fg}\; \end{array}\right.$$ (6)

$$J_{constant}=-J_{diffus,\;0}=D_{mFg}N_{mFg\;RBC,0}\left(\frac{\phi_{0}-1}{\phi_{0}}\right)/{\left(1.4\delta_{Fg}\right)}^{2}\;;\;\phi_{0}=\frac{N_{mFg\;RBC,0}}{N_{bFg}}$$ (7)

$$J_{diffus}={-D}_{mFg}\left(\frac{N_{mFg\;RBC}\;\phi_{0}-N_{mFg\;RBC,0}}{\phi_{0}}\right)/{\left(1.4\delta_{Fg}\right)}^{2}$$ (8)

where $J_{constant}$ and $J_{diffus}$ are the mFg sources on the RBC surface resulting from an assumed constant rate of recruitment and a normal diffusive flux following the bulk to surface concentration gradient. At initialization, the two sources cancel out each other to result in a zero net adsorption for the doublet in equilibrium (Eq. 7). $N_{mFg\;RBC,0}$ and $\phi_{0}$ are the surface concentration of mFg and surface to bulk concentration ratio of mFg at the initial equilibrium. $N_{bFg}$ is the bulk concentration of Fg in plasma. The expression 1.4 $\delta_{Fg}$ relates to the estimation of the adsoprtion layer which we adopted from the theoretical depletion layer thickness estimate (Vincent et al., 1986; Neu and Meiselman, 2002) since they are corollary concepts alluding to surface layer physiochemical dinstinction from the bulk solution.

### Intercellular friction model

To study the role of intercellular friction in aggregation mechanics, we defined a vertex to surface friction level which was mediated in our friction model by the intercellular gap plasma viscosity ( $\mu_{gap}$ ), glycocalyx height ( $r_{glyco}$ ) and the local separation distance between RBC surfaces ( $r_{sep}$ ). Intercellular friction, ${\vec{F}}_{fric,i}$ calculated on an RBC surface mesh vertex “i” in the intercellular space was determined by assuming a Couette flow profile between the two RBCs sliding in the doublet:

$${\vec{F}}_{fric,i}=-\mu_{gap}\left[\frac{{\vec{v}}_{ij}-\left({\vec{v}}_{ij}∙{\hat{e}}_{n,i}\right){\hat{e}}_{n,i}}{r_{sep}-2r_{glyco}}\right];\;{\vec{v}}_{ij}={\vec{v}}_{i}-{\vec{v}}_{j}$$ (9)

where ${\vec{v}}_{ij}$ , ${\vec{v}}_{i}$ and ${\vec{v}}_{j}$ are the relative velocity, local velocity at mesh vertex “i” on the RBC surface mesh and local velocity on the neighboring triangular mesh element “j” of the pairing RBC surface respectively; ${\hat{e}}_{n,i}$ is the local surface normal at mesh vertex “i”.

### Viscoelastic RBC model

For representation of RBC deformation mechanics, we employed the coarse-grained particle model (CGPM) developed by (Pivkin and Karniadakis, 2008; Fedosov et al., 2010). Membrane strain energy in the CGPM was calculated on a triangular surface mesh where constitutive expressions for areal deformation energy $U_{PM\;area}$ and bending energy $U_{PM\;bend}$ of the plasma membrane (PM), shearing energy $U_{CSK\;shear}$ of the viscoelastic RBC cytoskeleton (CSK) and compressive energy $U_{cytosol\;vol}$ of the incompressible cytosolic volume determined the internal forces ( ${\vec{F}}_{elas,i}$ ) of the membrane deformation at each nodal vertex ( i ) of the RBC mesh:

$${\vec{F}}_{elas,i}=-\frac{\partial U_{RBC,i}}{\partial {\vec{x}}_{i}};\;U_{RBC}=U_{CSK\;shear}+{U_{PMbend}+U}_{PM\;area}+U_{cytosol\;vol}\left(10\right)$$
where ${\vec{x}}_{i}$ is the positional vector for the RBC mesh vertex. For more details on the strain energy functions, please refer to section A in the supplementary materials.

In addition to the internal elastic forces, the rate of RBC deformation was modulated by the membrane viscosity. Membrane viscosity was represented by the dissipative particle formulation (Allen and Tildesley, 1987; Español, 1998; Fedosov et al., 2010):

$${\vec{F}}_{visc,ij}=-\eta^{T}{\vec{v}}_{ij}-\eta^{C}\left({\vec{v}}_{ij}\cdot {\hat{e}}_{ij}\right){\hat{e}}_{ij};\;\eta_{m}=\sqrt{3}\eta^{T}+\frac{{\sqrt{3}\eta }^{C}}{4};\;\eta^{C}=\frac{\eta^{T}}{3}\left(11\right)$$
where ${\vec{F}}_{visc,ij}$ is the dissipative force from membrane viscosity effects in the CSK, ${\vec{v}}_{ij}$ is the relative velocity between CSK mesh vertices i and j , ${\hat{e}}_{ij}$ is the unit displacement vector between i and j , $\eta^{T}$ and $\eta^{C}$ are dissipative coefficients related to the surface viscosity ( $\eta_{m}$ ) of the CSK.

Displacement of RBC mesh vertices was performed according to Newton’s second law motion:

$$d{\vec{x}}_{RBC,i}={\vec{v}}_{i}∆t;\;{\vec{v}}_{i}={\vec{v}}_{i,\;old}+d{\vec{v}}_{i};\;\;d{\vec{v}}_{i}=\frac{{\vec{F}}_{elas,i}+\sum_{j}{\vec{F}}_{visc,\;ij}+{\vec{F}}_{agg,i}+{\vec{F}}_{fric,i}}{m_{i}}∆t$$ (12)

where $d{\vec{x}}_{RBC,i}$ is displacement vector to update mesh vertex position, ∆t is the time step size for the numerical integration of Eq. 12, ${\vec{v}}_{i}$ is the newly updated vertex velocity and ${\vec{v}}_{i,\;old}$ is the vertex velocity from the previous time step. $d{\vec{v}}_{i}$ is the velocity increment based on the total forces acting on the vertex.

The simulation parameters defining RBC deformability are summarized in Table 1.

**TABLE 1:** Mechanical properties of the RBC employed in simulation models.

		Remarks on validation and formulation
Resting or zero-strain shear elastic modulus, E_s0_	6.54 μN∙m^−1^	Matched against uniaxial stretch test by optical tweezers (Suresh et al., 2005): see Supplementary Figure S2
Elastic bending modulus, E_b_	2.4 × 10^–19^J	Matched against aspiration and membrane buckling experiment (Evans, 1983): see Supplementary Figure S3A,B
Effective area compressibility modulus, K_0_ = 2E_s0_ + k_a_ + k_d_	0.432 N∙m^−1^	Matched against swollen RBC aspiration experiment (Evans et at., 1976 and Evans, 1989): see Supplementary Figure S3Ci
Global area compressibility coefficient, k_a_	0.288 N∙m^−1^	Formulation from Fedosov et al., 2010: see Supplementary Figure S3Ci, S3Cii
Local area compressibility coefficient, k_d_	0.144 N∙m^−1^	Formulation from Fedosov et al., 2010: see Supplementary Figure S3Ci, S3Cii
Volume correction penalty coefficient, k_Ω_	220 N∙m^−3^	Parameter value from Fedosov et al., 2010
Membrane viscosity, η_m_	0.7 μN∙s∙m^−1^	Matched against shape recovery time from large deformation uniaxial stretch tests (Hochmuth et al., 1979; Mills et al., 2004):see Supplementary Figure S4B

## Results and discussion

**FIGURE 3:**
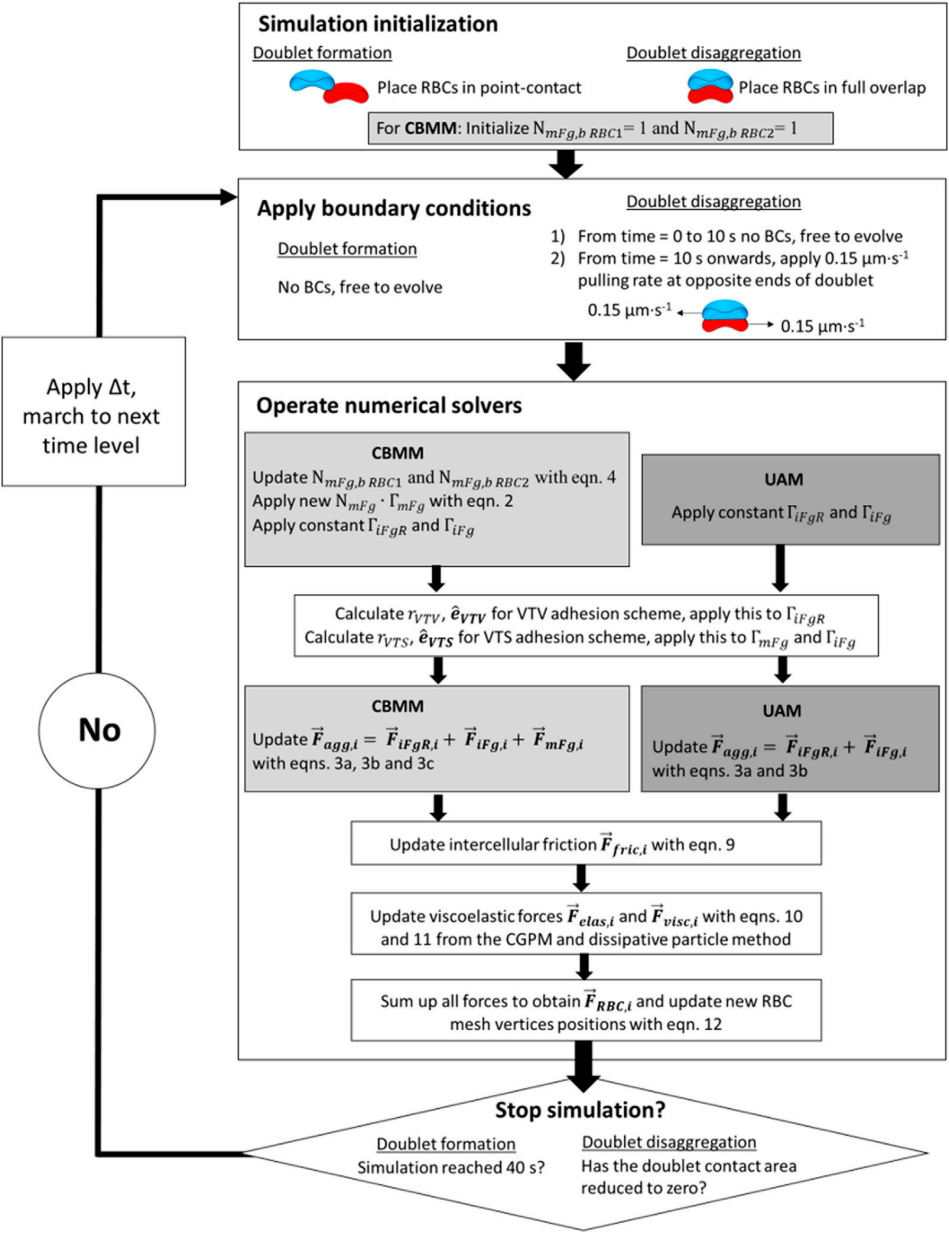
Block diagram for flow of simulation procedures in the aggregate formation simulations and doublet disaggregation simulations.

Following the construction of the CBMM and the UAM, we performed two types of simulations to study 1) the mechanics of cross-bridge types in RBC doublet formation and 2) the role of cross-bridge migration in doublet disaggregation. The sequence of procedures for these two simulations is summarized in Figure 3.

### Role of cross-bridge adhesion scheme and intercellular friction in modulating adhesion area for RBC doublet formation

At the two-cell aggregate (doublet) level, doublet formation in plasma undergoes three distinct phases of organization: the initial local contact phase, sliding phase of rapid contact area growth and the final creeping phase of slow contact growth (Dunlop et al., 1984). In reconstituted media containing only Fg and Alb, doublet formation under zero-flow conditions exhibited only the local contact phase (Lee et al., 2016b). We examined the case of adhesive contact area growth between initially point-contacted RBC pairs, in order to describe the cross-bridge scenarios that best match the kinematics observed in experiments (Dunlop et al., 1984; Khokhlova et al., 2012) and provide a mechanistic explanation of the plasma-constituent dependent aggregation. For this, we performed 5 groups of doublet formation simulations employing different permutations of the adhesion scheme. Each group employed three levels of total affinity $\Gamma_{affin}$ magnitude: 0.5, 1 and 1.5 μJ⋅m^−2^. Cross-bridging in group A contained only mobile non-specific cross-bridges (mFgB) and employed VTS adhesion. Group B consisted of only non-specific immobile cross-bridging (iFgB) and employed VTS adhesion. Group C represented a mixed cross-bridge scenario containing both mobile non-specific cross-bridges (mFgB) employing VTS and immobile specific cross-bridges (iFgRB) employing VTV. Group D represented another mixed cross-bridge scenario but with only immobile cross-bridges. The first fraction was the non-specific immobile cross-bridges (iFgB) prescribing VTS adhesion. The second fraction was specific cross-bridges (iFgRB) prescribing VTV adhesion. Group E consisted solely of specific cross-bridges (iFgRB) following VTV adhesion. A full description of simulation parameters defining the 15 sets of simulations can be found in Table 2.

**TABLE 2:** Simulation parameters for doublet formation simulations.

		Total starting bridge affinity (µJ·m^−2^]	Specific cross-bridge affinity (VTV) (µJ·m^−2^)	Immobile non-specific cross-bridge affinity (VTS) (µJ·m^−2^)	Mobile non-specific cross-bridge affinity (VTS) (µJ·m^−2^)	Starting mFg density on RBC1	Starting mFg density on RBC2	mFg diffusivity (m^2^·s^−1^)	mFg Asorption modulation	MP spatial decay coefficient (nm^−1^)	Zero-force separation distance (nm)	Cut-off separation distance (nm)	Glycocalyx height (nm)	Intercellular gap plasma viscosity (cP)	
Model	Presentation in paper	Γ_affin_	Γ_iFgR_	Γ_iFg_	Γ_mFg_	N_mFg RBC1,t0_	N_mFg RBC2,t0_	D_mFg_	on/off	β	r_0_	r_cutoff_	r_glyco_	µ_gap_	Representation of interaction type
CBMM with VTS	Group A (0.5 μJ·m^−2^) in Figure 3A and SM1, (0.5 μJ·m^−2^) in Figure 3B	0.5	0	0	0.25	1	1	1 × 10^–15^	off	0.05	20	100	5	3.6	non-specific mobile cross-bridge (mFgB)
CBMM with VTS	Group A (1.0 μJ·m^−2^) in Figure 3A and SM1, (1.0 μJ·m^−2^) in Figure 3B, and friction-optimized model in Figure 3C	1	0	0	0.5	1	1	1 × 10^–15^	off	0.05	20	100	5	3.6	non-specific mobile cross-bridge (mFgB)
CBMM with VTS	Group A (1.5 μJ·m^−2^) in Figure 3A and SM1, (1.5 μJ·m^−2^) in Figure 3B	1.5	0	0	0.75	1	1	1 × 10^–15^	off	0.05	20	100	5	3.6	non-specific mobile cross-bridge (mFgB)
UAM with VTS	Group B (0.5 μJ·m^−2^) in Figure 3A and SM1	0.5	0	0.5	0	0	0	N.A.	N.A.	0.05	20	100	5	3.6	non-specific immobile cross-bridge (iFgB)
UAM with VTS	Group B (1.0 μJ·m^−2^) in Figure 3A and SM1	1	0	1	0	0	0	N.A.	N.A.	0.05	20	100	5	3.6	non-specific immobile cross-bridge (iFgB)
UAM with VTS	Group B (1.5 μJ·m^−2^) in Figure 3A and SM1	1.5	0	1.5	0	0	0	N.A.	N.A.	0.05	20	100	5	3.6	non-specific immobile cross-bridge (iFgB)
CBMM with VTS + iFgRB VTV	Group C (0.5 μJ·m^−2^) in Figure 3A and SM1	0.5	0.25	0	0.125	1	1	1 × 10^–15^	off	0.05	20	100	5	3.6	50% non-specific mobile cross-bridge (mFgB) 50% specific cross-bridges (iFgRB)
CBMM with VTS + iFgRB VTV	Group C (1.0 μJ·m^−2^) in Figure 3A and SM1	1	0.5	0	0.25	1	1	1 × 10^–15^	off	0.05	20	100	5	3.6	50% non-specific mobile cross-bridge (mFgB) 50% specific cross-bridges (iFgRB)
CBMM with VTS + iFgRB VTV	Group C (1.5 μJ·m^−2^) in Figure 3A and SM1	1.5	0.5	0	0.5	1	1	1 × 10^–15^	off	0.05	20	100	5	3.6	66% non-specific mobile cross-bridge (mFgB) 33% specific cross-bridges (iFgRB)
UAM with VTS and VTV	Group D (0.5 μJ·m^−2^) in Figure 3A and SM1	0.5	0.25	0.25	0	0	0	N.A.	N.A.	0.05	20	100	5	3.6	50% non-specific immobile cross-bridge (iFgB) 50% specific cross-bridges (iFgRB)
UAM with VTS and VTV	Group D (1.0 μJ·m^−2^) in Figure 3A and SM1	1	0.5	0.5	0	0	0	N.A.	N.A.	0.05	20	100	5	3.6	50% non-specific immobile cross-bridge (mFgB) 50% specific cross-bridges (iFgRB)
UAM with VTS and VTV	Group D (1.5 μJ·m^−2^) in Figure 3A and SM1	1.5	0.5	1	0	0	0	N.A.	N.A.	0.05	20	100	5	3.6	66% non-specific immobile cross-bridge (mFgB) 33% specific cross-bridges (iFgRB)
UAM with VTV	Group E (0.5 μJ·m^−2^) in Figure 3A and SM1	0.5	0.5	0	0	0	0	N.A.	N.A.	0.05	20	100	5	3.6	specific cross-bridge (iFgRB)
UAM with VTV	Group E (1.0 μJ·m^−2^) in Figure 3A and SM1	1	1	0	0	0	0	N.A.	N.A.	0.05	20	100	5	3.6	specific cross-bridge (iFgRB)
UAM with VTV	Group E (1.5 μJ·m^−2^) in Figure 3A and SM1	1.5	1.5	0	0	0	0	N.A.	N.A.	0.05	20	100	5	3.6	specific cross-bridge (iFgRB)
UAM with VTS	Low-friction model in Figure 3C	1	0	1	0	0	0	N.A.	N.A.	0.05	20	100	0	1.8	non-specific immobile cross-bridge (iFgB)
UAM with VTS	Long-range MP model in Figure 3C	1	0	1	0	1	0	N.A.	N.A.	0.05	500	2000	0	1.8	non-specific immobile cross-bridge (iFgB)

**FIGURE 4:**
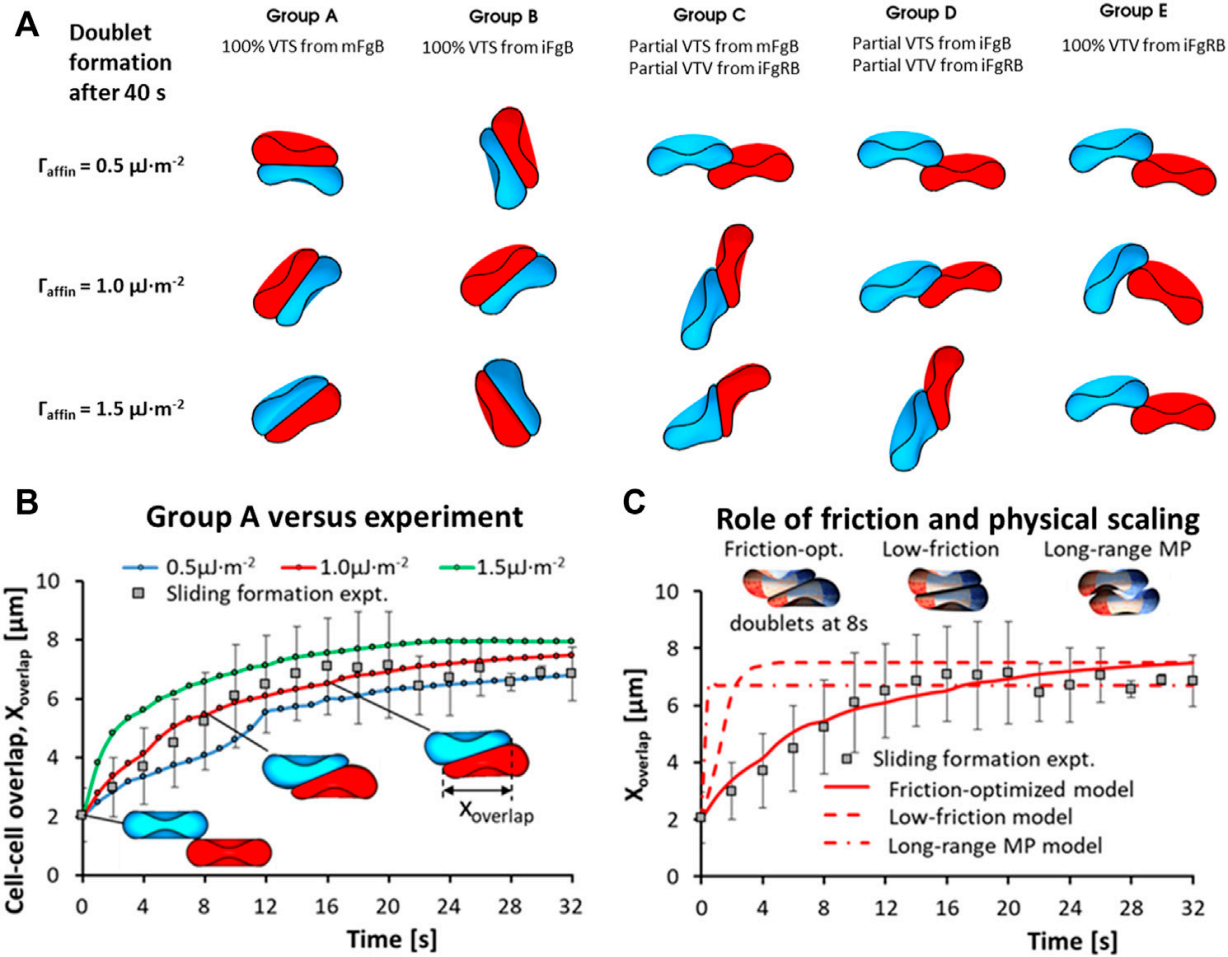
RBC-doublet formation kinematics simulations. **(A)** Simulation results of the doublet formation equilibrium at 40 s after initial point contact, under various adhesion affinity $\Gamma_{affin}$ permutations (*Simulation movie in SM1 and SM2*); **(B)** Correspondence between simulation models and experiments (Khokhlova et al., 2012) for the VTS adhesion scheme employed in Group A models within the $\Gamma_{affin}$ range 0.5–1.5 μJ⋅m^−2^, an intercellular gap separation of 20 nm and gap plasma viscosity of 3.6 cP; **(C)** Effect of intercellular friction and physical scaling of interaction the length-scale on the doublet formation rate for the $\Gamma_{affin}$ = 1 μJ⋅m^−2^ model (*Simulation movie in SM3*).

Figure 4A shows the results of our doublet formation models for the 5 groups (simulation movie of this result can be seen in SM1). Groups employing any permutation of the VTV scheme saw strong adhesive locking where the conjugation of pairing adhesion vertices between the two cells restricted further locomotion between RBCs. Consequently, groups C, D and E displayed no spontaneous increase in the cell overlap area beyond the initial point contact. The failure to generate spontaneous adhesion area growth from VTV arose due to the distance between adhesion vertices (receptors) naturally exceeding the $r_{cutoff}$ . Our adhesion model employs ∼5,000 adhesion vertices at which the distance-based MP attraction/repulsion can be defined. We based this vertex density on the Fg-receptor density in RBCs (Lominadze and Dean, 2002). Based on the vertex density the average distance between adhesion vertices ( $r_{v}$ ) on each RBC is ∼200 nm. We employed $r_{cutoff}$ = 100 nm and this meant that the VTV was severely limited in receptor availability for cross-bridge recruitment and spontaneous adhesion area growth (Figure 1D). These VTV models matched the formation kinematics in the experiment with doublets suspended in PBS media with physiological concentrations of Fg and Alb (Lee et al., 2016b). In that experiment, RBC pairs in point contact did not spontaneously slide to form larger doublet contact area. Interpreting our VTV simulation findings, we hypothesize a strong propensity for Fg and Alb to form specific cross-bridges between aggregating RBCs when other plasma proteins are absent.

In contrast to Groups C to E, Groups A and B with pure VTS schemes showed the sliding and creeping stages of doublet contact area increase. Essentially, the VTS results matched the experimental doublet formation kinematics in plasma where sliding and adhesion contact area increase has been observed (Khokhlova et al., 2012). It may be that the presence of other blood proteins in plasma appears to shift the dominant cross-bridging mechanism towards weaker non-specific cross-bridging mechanisms. In summary, non-specific interactions iFgB and mFgB were found to promote spontaneous adhesion area growth during doublet formation.

Next, we studied the role of intercellular friction which was mediated in our RBC interaction simulation by the intercellular gap plasma viscosity ( $\mu_{gap}$ ), glycocalyx height ( $r_{glyco}$ ) and the local separation distance between RBC surfaces ( $r_{sep}$ ). As shown in Figure 4B (simulation movie in SM2) and Figure 4C (simulation movie in SM3), sliding formation kinematics match experiments (Khokhlova et al., 2012) for the VTS schemes with a friction-optimized model ( $r_{cutoff}$ = 100 nm, $r_{0}$ = 20 nm (Chien, 1973), $r_{glyco}$ = 5 nm (Skutelsky et al., 1977; Suganuma et al., 1985), $\mu_{gap}$ = 3.6 cP); the average rates of formation to reach 80% of final overlap were 0.18, 0.28 and 0.36 μm⋅s^−1^ for the employed $\Gamma_{affin}$ cases of 0.5, 1 and 1.5 μJ⋅m^−2^. In comparison, a mean rate of formation in plasma has been experimentally determined to be ∼0.3 μm⋅s^−1^ (Dunlop et al., 1984; Khokhlova et al., 2012). Simulation sets performed with the low-friction model ( $r_{cutoff}$ = 100 nm, $r_{0}=20\;nm$ , $r_{glyco}=0\;nm$ , ${\;\mu }_{gap}=1.8\;cP$ ) and long-range MP model ( $r_{cutoff}$ = 2000 nm, $r_{0}=500\;nm$ , $r_{glyco}=0\;nm$ , ${\;\mu }_{gap}=1.8\;cP$ ) showed exaggerated doublet formation rates beyond the physiological rates observed in the experiments, thus highlighting the importance of correct length and dynamic scaling in the aggregation model mechanics in order to dynamically match the empirical observations. It is of note that the bulk of RBC aggregation models employed in blood flow transport models have been employing attraction length scales similar to the long-range MP model tested here and it is likely that these models have been overexaggerating the formation speed of aggregates in their models (Liu and Liu, 2006; Zhang et al., 2008; Wang et al., 2009; Zhang et al., 2009; Fedosov et al., 2011a; Fedosov et al., 2011b; Xu et al., 2013; Yin et al., 2013; Li et al., 2014; Wang and Xing, 2014; Ye et al., 2014a; b; Katanov et al., 2015; Ye and Peng, 2019; Ye et al., 2019). This may affect the rheological representation of RBC aggregation at low shear rates where RBC aggregation increases blood viscosity. On a secondary note, the plasma viscosity in the intercellular gap has been predicted to be higher than bulk plasma viscosity levels in our friction-optimized model. This could be due to high Fg concentration in the intercellular space and the augmented levels of inter-protein interaction in a confined environment that serve to limit Fg diffusivity and raise solution viscosity (Zuev et al., 2017)—this has been demonstrated for protein solutions of high protein concentration and high degree of protein-cluster formations (von Bülow et al., 2019).

A full description of simulation parameters for the models studying frictional effects on doublet formation kinematics can be found in Table 2.

### Conditional validity of the cross-bridge migration hypothesis for RBC doublet disaggregation in plasma

In the preceding results section, we demonstrated the VTS scheme representing iFgB and mFgB to be more representative of RBC doublet formation kinematics in blood plasma and from here on we only employ the VTS. Here, we will discuss the parametric optimization required for the cross-bridge migration model (CBMM) to support experiment. We will also highlight the deficiencies of the uniform affinity model (UAM) that lacks the critical adhesion strength modulation feature required to match experiments. To compare the two models, we followed the experimental protocol for doublet disaggregation with optical tweezers (OT) where the distal ends of each RBC were displaced in opposite directions at a fixed rate of 0.15 μm∙s^−1^. The simulated trap tension to doublet displacement profile was compared against the tension-displacement behavior in the OT experiment prior to trap escape tension at 29 ± 3 pN. The mechanics of doublet disaggregation in the OT experiment can be understood as a force-balance between the component of adhesion force, $F_{agg,x}$ along the pulling axis and the trap tension (or disaggregation force), $F_{OT}$ .

**FIGURE 5:**
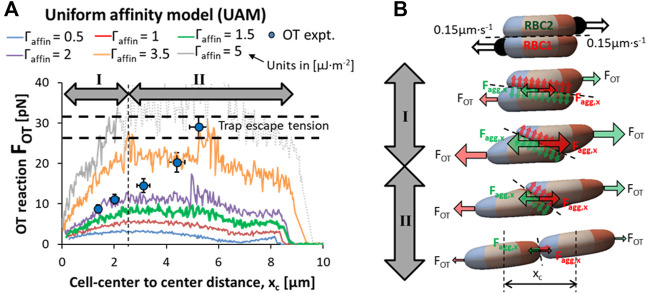
Failure of the UAM to recapitulate optical tweezers (OT) experiment results. **(A)** Comparison of the force-displacement response in OT-driven doublet disaggregation predicted by the uniform affinity model (UAM) against the experimental results highlights the failure of UAM to match experimental phenomenon (*simulation movie in SM4*) **(B)** A force-balance schematic of the RBC doublet disaggregation with OT showing the simultaneous reorientation of the adhesion plane and contact area loss contributing to the increasing tension stage (stage I) and reducing tension stage (stage II) of the disaggregation.

Doublet disaggregation simulations employing the UAM (see simulation movie in SM4) obtained disaggregation force trends that were a poor match with the empirical results (Khokhlova et al., 2012). A consistent two-stage trend was observed in the force-displacement curve in the UAM performed with $\Gamma_{affin}$ range of 0.5–5 μJ⋅m^−2^. In the initial stage of disaggregation (stage I in Figures 5A,B), a reorientation of the adhesion interface under membrane tension increased $F_{agg,x}$ (see force-diagram in Figure 5A) and caused an initial rise in $F_{OT}$ . This was followed by a stage of steady decrease in $F_{OT}$ as the effect of contact area loss on $F_{agg,x}$ exceeded the effect of the interface reorientation (stage II in Figures 5A,B). Finally, an abrupt drop of the disaggregation force to zero indicated the final displacement point at which the doublet undergoes complete disaggregation. Strikingly, the UAM results are neither a quantitative nor qualitative match for the experiment, for which doublet tension exceeded the trap escape tension of 29 ± 3 pN while still exhibiting a stage I trend. Thus, in addition to the interface reorientation which has a limited effect in augmenting $F_{agg,x}$ and $F_{OT}$ , we posit that the additional mechanism required for continued increase in $F_{agg,x}$ comes through the accumulation of cross-bridges in the diminishing contact region.

**FIGURE 6:**
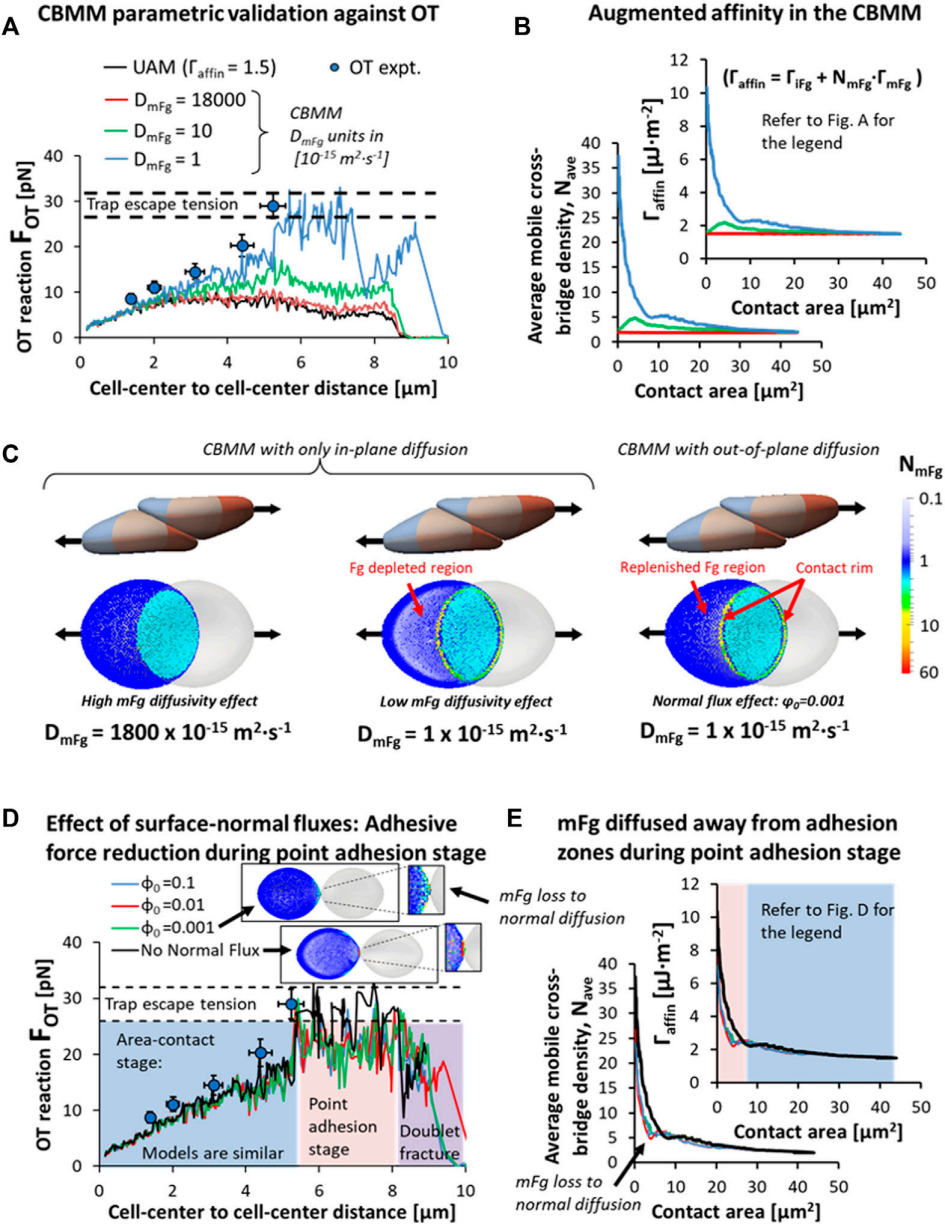
Validation of CBMM against RBC-doublet disaggregation optical tweezers (OT) experiments. **(A)** Comparison of the cross-bridge migration model (CBMM) under varying mFg diffusivity, DmFg [1x10^−15^ m^2^∙s-1] against the experimental results. **(B)** Augmented surface affinity of the RBC doublet adhesion due to an increase in the average cross-bridge density in the intercellular gap prescribed by convection-diffusion mechanisms in the CBMM. **(C)** Distribution maps of surface-adsorbed Fg during doublet disaggregation predicted by the parametric simulation study. High mFg diffusivity effect: Fast lateral diffusion of mFg ensured homogenous mFg distribution. Low mFg diffusivity effect: Slow rate of mFg diffusion allowed frictional drift to accumulate mFg in contact rim; Normal flux effect: Depleted regions were replenished by out-of-plane diffusion (Simulation movie in SM5). **(D,E)** Models with surface-normal diffusion under varying resting bulk to surface concentration ratios ( $\phi_{0}$ ) indicated mFg loss and adhesion weakening only during point-adhesion stage, prior to doublet fracture (Simulation movie in SM6).

Applying our CBMM to the same OT protocol for dissociating doublets, we found the level of cross-bridge accumulation required for an increase in adhesivity matching the experiment (Khokhlova et al., 2012) was achieved when the diffusivity of the surface-adsorbed mobile Fg ( $D_{mFg}$ ) was < 1 × 10^−15^ m^2^⋅s^−1^ (Figure 6A). As a basis for comparison, the model employing $D_{mFg}$ based on the Fg diffusivity in aqueous solution ( $1.8x{10\;}^{-11}$ m^2^⋅s^−1^ (Larsson et al., 1987)) predicted a negligible cross-bridge accumulation rate (red curve in Figure 6B) and a force-displacement result close to the result predicted by the UAM with $\Gamma_{affin}$ = 1.5 μJ⋅m^−2^ (red vs. black curve in Figure 6A). This was because the fast lateral diffusion of mFg ensured homogenous mFg distribution on RBC surfaces, thereby providing little augmentation to the cross-bridge recruitment and adhesion magnitude (Figure 6C). Note that in our comparison between the CBMM and the UAM, the total affinity is the same at simulation initialization: $\Gamma_{affin}$ for the CBMM in the intercellular gap as given by Eq. 2 was 1.5 μJ⋅m^−2^ ( $\Gamma_{iFg}$ = 1 μJ⋅m^−2^ & $\Gamma_{mFg}$ = 0.25 μJ⋅m^−2^) since $N_{mFg\;RBC1}$ and $N_{mFg\;RBC2}$ were both set to 1 at the initialization. As shown in Figure 6B, reducing the $D_{mFg}$ permitted a greater accumulation of Fg cross-bridges as indicated by the increase in average bridge density $N_{ave}$ in the intercellular gap. With this diffusivity setting, friction-induced accumulation of mFg in contact rims of the intercellular contact region could persist throughout the entire doublet disaggregation as the diffusion transport of mFg now occurred at timescales beyond the time required to fully disaggregate the doublet. Consequently, the rise in the disaggregation force required to separate the doublet resulted from the increase in average bridge density $N_{ave}$ and the corresponding increase in $\Gamma_{affin}$ . Correspondingly, we can observe with the mFg distribution maps in Figure 6C the mutually competing effects of diffusion (prevents accumulation) against friction-induced mFg drift (promotes accumulation) in the intercellular space for RBC doublets undergoing shear-induced disaggregation.

The $D_{mFg}$ setting utilized in the optimized CBMM matching the OT experiment suggests that the diffusivity of surface-adsorbed mFg may be considerably lower than its aqueous counterpart Fg. This may be a result of the intermolecular attraction between Fg molecules and the added presence of RBC-bound Fg (specifically bound to RBC receptors) in the narrow intercellular region; it may be that mFg is a large multi-protein Fg-complex with considerably reduced diffusivity. Such physiochemical interactions under confinement scenario should limit the mobility of the mFg on the RBC surface as similarly reported in the case of high Fg concentration conditions (Zuev et al., 2017). Furthermore, we have not explicitly modeled the reaction kinetics of cross-bridge formation and cleavage in the mFg transport equation (Eq. 4) as there is virtually no experimental data and as such its parametric inclusion in Eq. 4 would be extremely difficult in our CBMM. Consequently, the phenomenological reflection of bridge formation and cleavage kinetics to mFg transport in our present CBMM is through the reduction of the effective diffusivity of mFg–this may be another probable reason for the significantly low $D_{mFg}$ predicted in our present CBMM.

We also examined the role of normal fluxes and bulk to surface concentration gradients through the ${\dot{S}}_{adsorp}$ term in Eq. 4 and requisite parameters in Eqs. 6–8. As seen from Figure 6D, a comparison of the CBMM with zero ${\dot{S}}_{adsorp}$ (black curves) against the CBMM considering ${\dot{S}}_{adsorp}$ with $\phi_{0}=0.001-0.1$ shows that the effect of adsorption flux and bulk concentration gradients in changing the doublet disaggregation force and CBMM-predicted affinity augmentation was marginal during the area-contact stage of the doublet disaggregation (highlighted blue area in graphs in Figure 6D,E). During this stage, the primary change in surface phenomena elicited by the normal flux consideration was a replenishing of mFg in the mFg-depleted regions where mFg had been dragged away into the intercellular adhesion space (Figure 6C and simulation movie in SM5). Compared to the zero ${\dot{S}}_{adsorp}$ model, the ${\dot{S}}_{adsorp}$ model predicted a surface concentration of mFg in these regions closer to the initialized concentration of 1. The bridge accumulation phenomenon in the intercellular adhesion space was largely unaffected by the ${\dot{S}}_{adsorp}$ consideration in the CBMM during the area-contact stage (Figure 6E). However, as the doublet disaggregation progressed, point-adhesion and necking of the membrane regions around the adhesion interface exposed the mFg bridges to the strong surface-normal concentration gradient which resulted in losses of mFg to the bulk environment *via* diffusion for the CBMM with $\phi_{0}=0.001-0.1$ models (see Figure 6E and inset figure in Figure 6D). Despite the loss in adhesivity level during the point-adhesion stage, there was no difference between the zero ${\dot{S}}_{adsorp}$ model and the $\phi_{0}=0.001-0.1$ models for the prediction of trap escape as all models tested in Figure 6D,E reached the 29 ± 3 pN threshold reported in the OT experiments.

A summary of the simulations performed in this section and their parameter settings can be found in Table 3.

**TABLE 3:** Simulation parameters for CBMM and UAM in doublet disaggregation simulations.

		Total starting bridge affinity (µJ·m^−2^)	Specific cross-bridge affinity (VTV) (µJ·m^−2^)	Immobile non-specific cross-bridge affinity (VTS) (µJ·m^−2^)	Mobile non-specific cross-bridge affinity (VTS) [µJ·m^−2^)	Starting mFg density on RBC1	Starting mFg density on RBC2	mFg diffusivity [m^2^·s^−1^)	mFg Asorption modulation	Surface to bulk mFg ratio at equilibirum	MP spatial decay coefficient (nm^−1^)	Zero-force separation distance (nm)	Cut-off separation distance (nm)	Glycocalyx height (nm)	Intercellular gap plasma viscosity (cP)	
Model	Presentation in paper	Γ_affin_	Γ_iFgR_	Γ_iFg_	Γ_mFg_	N_mFg RBC1,t0_	N_mFg RBC2,t0_	D_mFg_	on/off	φ_0_	β	r_0_	r_cutoff_	r_glyco_	µ_gap_	Representation of interaction type
UAM with VTS	UAM (0.5 μJ·m^−2^) in Figure 4A and SM4	0.5	0	0.5	0	0	0	N.A.	N.A.	N.A.	0.05	20	100	5	3.6	non-specific immobile cross-bridge (iFgB)
UAM with VTS	UAM (1.0 μJ·m^−2^) in Figure 4A and SM4	1	0	1	0	0	0	N.A.	N.A.	N.A.	0.05	20	100	5	3.6	non-specific immobile cross-bridge (iFgB)
UAM with VTS	UAM (1.5 μJ·m^−2^) in Figure 4A, Figure 5A and SM4	1.5	0	1.5	0	0	0	N.A.	N.A.	N.A.	0.05	20	100	5	3.6	non-specific immobile cross-bridge (iFgB)
UAM with VTS	UAM (2.0 μJ·m^−2^) in Figure 4A and SM4	2	0	2	0	0	0	N.A.	N.A.	N.A.	0.05	20	100	5	3.6	non-specific immobile cross-bridge (iFgB)
UAM with VTS	UAM (3.5 μJ·m^−2^) in Figure 4A and SM4	3.5	0	3.5	0	0	0	N.A.	N.A.	N.A.	0.05	20	100	5	3.6	non-specific immobile cross-bridge (iFgB)
UAM with VTS	UAM (5.0 μJ·m^−2^) in Figure 4A and SM4	5	0	5	0	0	0	N.A.	N.A.	N.A.	0.05	20	100	5	3.6	non-specific immobile cross-bridge (iFgB)
CBMM with VTS	High diffusivity (1800 × 10^–15^ m^2^·s^−1^) in Figures 5A–C and SM5	1.5	0	1	0.25	1	1	1.8 × 10^–12^	off	N.A.	0.05	20	100	5	3.6	non-specific cross-bridge (immobile iFgB and mobile mFgB)
CBMM with VTS	Moderate diffusivity (10 × 10^–15^ m^2^·s^−1^) in Figures 5A,B	1.5	0	1	0.25	1	1	1 × 10^–14^	off	N.A.	0.05	20	100	5	3.6	non-specific cross-bridge (immobile iFgB and mobile mFgB)
CBMM with VTS	Low diffusivity (1 × 10^–15^ m^2^·s^−1^) in Figures 5A–C and no normal flux case in Figures 5D,E, and SM5	1.5	0	1	0.25	1	1	1 × 10^–15^	off	N.A.	0.05	20	100	5	3.6	non-specific cross-bridge (immobile iFgB and mobile mFgB)
CBMM with VTS	Bulk to surface flux effect model (φ_0_ = 0.001) in Figures 5C–E, SM5 and SM6	1.5	0	1	0.25	1	1	1 × 10^–15^	on	0.001	0.05	20	100	5	3.6	non-specific cross-bridge (immobile iFgB and mobile mFgB)
CBMM with VTS	Bulk to surface flux effect model (φ_0_ = 0.01) in Figures 5D,E and SM6	1.5	0	1	0.25	1	1	1 × 10^–15^	on	0.01	0.05	20	100	5	3.6	non-specific cross-bridge (immobile iFgB and mobile mFgB)
CBMM with VTS	Bulk to surface flux effect model (φ_0_ = 0.1) in Figures 5D,E and SM6	1.5	0	1	0.25	1	1	1 × 10^–15^	on	0.1	0.05	20	100	5	3.6	non-specific cross-bridge (immobile iFgB and mobile mFgB)
CBMM with VTS	Bulk to surface flux effect model (φ_0_ = 2) in SM6	1.5	0	1	0.25	1	1	1 × 10^–15^	on	2	0.05	20	100	5	3.6	non-specific cross-bridge (immobile iFgB and mobile mFgB)
CBMM with VTS	Bulk to surface flux effect model (φ_0_ = 10) in SM6	1.5	0	1	0.25	1	1	1 × 10^–15^	on	10	0.05	20	100	5	3.6	non-specific cross-bridge (immobile iFgB and mobile mFgB)

### Representation of pathophysiological aggregability ranges within the CBMM

With the baseline CBMM parameters ( $D_{mFg}$ = 1 × 10^−15^ m^2^⋅s^−1^) and intercellular frictional parameters ( $r_{cutoff}$ = 100 nm, $r_{0}$ = 20 nm, $r_{glyco}$ = 5 nm, $\mu_{gap}$ = 3.6 cP) for representation of the referenced aggregation behavior for RBC doublets in the OT disaggregation set up, we examined the case of RBC hyper-aggregation in systemic lupus erythematosus (SLE). For this pathophysiological study, we prescribed the assumed changes in surface concentration of Fg in our CBMM as an increase from the reference surface concentration ( $N_{mFg,b}$ ) of Fg by a factor of $N^{*}$ at equilibrium, where $N_{mFg,b}$ represents the lower physiological limit for bulk concentration of Fg. Additionally, pathophysiological aggregation promoted by alteration of the adhesion affinity are represented as an increase over reference affinity levels by a factor of $\Gamma^{*}$ , where $\Gamma^{*}$ = 1 represents the affinity factor for physiological aggregation. The reference affinity under normal aggregation conditions in healthy samples was set to $\left(\Gamma_{mFg,b},\;\Gamma_{iFg}\right)$ = (0.25, 1) μJ⋅m^−2^ as evaluated in the preceding section with the baseline physiological CBMM. With these considerations, Eq. 2 was recast in the following form:

$$\Gamma_{affin}=\Gamma_{iFg}+\left(N^{*}∙N_{mFg,b}\right)∙\left(\Gamma^{*}∙\Gamma_{mFg,b}\right);\;N_{mFG,\;b}=N_{mFg,b\;RBC1}+N_{mFg,b\;RBC2}$$ (13)

Following the baseline CBMM established in the earlier section, the baseline concentrations of mFg on RBC1 and RBC2 at equilibrium before doublet disaggregation was initiated were set at $N_{mFg,b\;RBC1}$ = 1 and $N_{mFg,b\;RBC2}$ = 1, hence $N_{mFG,\;b}$ = 2.

**FIGURE 7:**
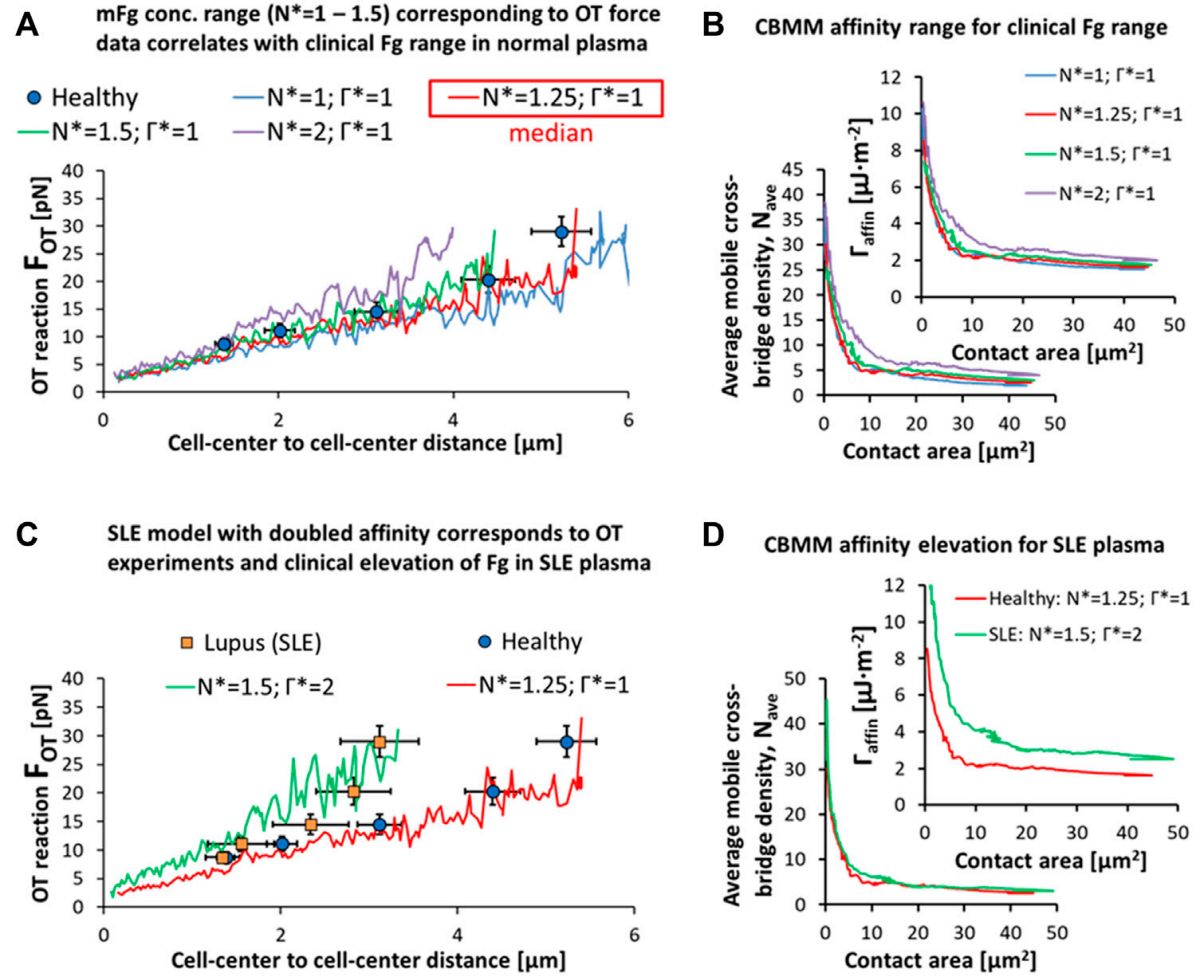
Pathophysiological representations of RBC doublet disaggregation. **(A,B)** Variation in the OT experimental data can be explained in the CBMM by variation in the surface adsorption of Fg in accordance with the clinical variation in Fg levels in plasma from healthy samples. **(C,D)** SLE samples results in OT can be explained by a dual-effect of elevation in surface adsorbed Fg (N* = 1.25 for median level in healthy RBCs and plasma to N* = 1.5 for median level in SLE RBCs in SLE plasma) and an increase in mFg mediated affinity (Γ* = 1 for healthy to Γ* = 2 in SLE). Simulation movies of the parametric variations in N* and Γ* can be seen in SM7.

First, we set out to describe the apparent relation between bulk concentration of Fg in plasma and the surface concentration of adsorbed Fg for doublets and plasma from healthy patients. There appeared to be a direct correspondence between the clinical range for systemic Fg levels in normal plasma from healthy samples and the surface concentration range of adsorbed Fg in the CBMM employed to fit the empirical variation in OT doublet disaggregation forces (Figure 7A). From the lower fit (blue curve in Figure 7A: $N^{*}$ = 1) to the higher fit (green curve in Figure 7A: $N^{*}$ = 1.5), a 50% increase in surface concentration (starting $N_{ave}$ increased from 2 to 3 in Figure 7A and Table 4) matches the ∼50% increase in bulk concentration of Fg from the lower physiological limit to the higher physiological limit in a healthy population (Fg bulk conc. in mg/dL: 243–357 for boys aged 4–14; 213–317 for men aged 20–30; 240–358 for men aged 40–50; 231–331 for women aged 40–50) (Tarallo et al., 1992).

**TABLE 4:** Simulation parameters for CBMM investigation of pathophysiological aggregation.

		Total starting bridge affinity (µJ·m^−2^)	Specific cross-bridge affinity (VTV) (µJ·m^−2^)	Immobile non-specific cross-bridge affinity (VTS) (µJ·m^−2^)	mFg affinity multiplier	Mobile non-specific cross-bridge affinity (VTS) (µJ·m^−2^)	Total starting mFg density	Conc. multiplier	Starting mFg density on RBC1	Starting mFg density on RBC2	mFg diffusivity (m^2^·s^−1^)	mFg Asorption modulation	Surface to bulk mFg ratio at equilibirum	MP spatial decay coefficient (nm^−1^)	Zero-force separation distance (nm)	Cut-off separation distance (nm)	Glycocalyx height (nm)	Intercellular gap plasma viscosity (cP)	
Model	Presentation in paper	Γ_affin_	Γ_iFgR_	Γ_iFg_	Γ*	Γ_mFg_	N_ave,t0_	N*	N_mFg,b RBC1,t0_	N_mFg,b RBC2,t0_	D_mFg_	on/off	φ_0_	β	r_0_	r_cutoff_	r_glyco_	µ_gap_	Representation of interaction type
CBMM with VTS	N* = 1; Γ* = 1 model in Figures 6A,B, healthy lower limit	1.5	0	1	1	0.25	2	1	1	1	1 × 10^–15^	on	0.001	0.05	20	100	5	3.6	non-specific cross-bridge (immobile iFgB and mobile mFgB)
CBMM with VTS	N* = 1.25; Γ* = 1 model in Figures 6A–D, healthy median match	1.625	0	1	1	0.25	2.5	1.25	1	1	1 × 10^–15^	on	0.001	0.05	20	100	5	3.6	non-specific cross-bridge (immobile iFgB and mobile mFgB)
CBMM with VTS	N* = 1.5; Γ* = 1 model in Figures 6A,B, healthy upper limit	1.75	0	1	1	0.25	3	1.5	1	1	1 × 10^–15^	on	0.001	0.05	20	100	5	3.6	non-specific cross-bridge (immobile iFgB and mobile mFgB)
CBMM with VTS	N* = 2; Γ* = 1 model in Figures 6A,B	2	0	1	1	0.25	4	2	1	1	1 × 10^–15^	on	0.001	0.05	20	100	5	3.6	non-specific cross-bridge (immobile iFgB and mobile mFgB)
CBMM with VTS	N* = 1.5; Γ* = 2 model in Figures 6C,D, Lupus (SLE) median match	2.5	0	1	2	0.25	3	1.5	1	1	1 × 10^–15^	on	0.001	0.05	20	100	5	3.6	non-specific cross-bridge (immobile iFgB and mobile mFgB)

Similarly, the hyper-stabilized state of the RBC doublet in SLE can be explained by an increase in the surface adsorption rate and surface concentration of bridging proteins like Fg in response to the increased levels of the proteins in blood plasma. The SLE doublet hyper-stability data in the disaggregation experiment was matched to a 20% increase in the surface concentration of Fg in the CBMM ( $N^{*}$ = 1.25 for median level in normal plasma to $N^{*}$ = 1.5 for median level in SLE plasma; Figure 7C) in addition to a doubling of the mFg mediated affinity ( $\Gamma^{*}$ = 1 for normal plasma to $\Gamma^{*}$ = 2 in SLE plasma). As shown in Figure 7D and by calculation of Eq. 13, the starting mFg concentration density ( $N_{ave}$ ) of the median healthy aggregation scenario was 2.5 and initial $\Gamma_{affin}$ before disaggregation initiation and bridge movement was 1.625 μJ⋅m^−2^. In the median SLE condition, the starting $N_{ave}$ was 3 and intial $\Gamma_{affin}$ before disaggregation initiation and bridge movement was 2.5 μJ⋅m^−2^. The predicted increase in surface concentration of Fg in the SLE model is comparable to the ∼30% increase (Ames et al., 2000) in bulk Fg concentrations for SLE patients. However, the doubling of the mFg-mediated surface affinity in order to match OT data on SLE doublet disaggregation suggests that there may be physiochemical alterations to the SLE RBC surface. Additionally, we also expect the modest rise in the plasma viscosity for SLE samples resulting from the Fg elevation in the plasma (Hazelton et al., 1985; Reid and De Ceulaer, 1999; Rosenson et al., 2001; Booth et al., 2007) to futher reduce $D_{mFg}$ in SLE RBC aggregate disaggregation dynamics thereby promoting greater cross-bridge accumulation during aggregate disaggregation.

A summary of the simulations performed in this section and their parameter settings can be found in Table 4.

### CBMM limitations

Because the elementary contributors to the inter-RBC interactions are diverse in plasma, we can only provide a framework of the cross-bridge migration hypothesis around Fg. The present CBMM therefore does not explicitly model the roles of other blood proteins which may play integral roles in mediating the affinity of Fg with the RBC surface or directly contribute to aggregation as demonstrated with serum-mediated RBC aggregation (Lee et al., 2016a). Despite this, our investigation into the underlying cross-bridging mechanisms in doublet formation with VTV and VTS adhesion schemes has been useful in demonstrating the doublet formation kinematics resulting from specific cross-bridges (iFgRB) *versus* non-specific cross-bridges (mFgB and iFgB). Beyond this, the model cannot elucidate on a deeper theoretical level the mechanistic interactions of other blood factors with Fg and how they may regulate the equilibrium levels of iFgR, iFgB and mFgB. For further development, we may prescribe the contributions of other blood factors on the action of the cross-bridging elements by transforming the adhesion energy potential, CBMM transport characteristics and employing a mixed scheme approach with both VTS and VTV conditionally present in the aggregation model. These further developments can only be done when the CFS experiments measuring the kinematics and force characteristics contributed by other plasma factors have been performed.

Another key aspect missing from the present CBMM is a consideration of a time-dependent cross-bridge formation which has been suggested to be on the order of seconds (Bronkhorst et al., 1997). We have assumed in our CBMM that both specific cross-bridging scenarios (iFgR) and non-specific cross-bridging scenarios iFgB and mFgB to be spontaneous and invariant to the timescales considered in our study. While the CBMM has been parametrically tweaked to match the empirical data, this does not suggest our time-invariance assumption to be true. For example, if we represented the surface affinity of a mobile bridging unit ( $\Gamma_{mFg}$ ) in the model to increase with time, the optimal value of $D_{mFg}$ required to produce a disaggregation force trend matching the experiment is expected to increase. Furthermore, specific cross-bridging mechanisms (iFgRB) may occur with increasing probability with time. Moreover, membrane fluctuation may overcome the unfavorable scenario of low receptor density and promote iFgRB, particularly in pathological scenarios such as SLE. Although this is outside of our present study scope due to the dearth of experimental data, a stochastic consideration of temporal and spatial factors regulating specific cross-bridging may provide a more accurate theory of the plasma-mediated RBC aggregation mechanism.

Finally, while this is not a direct limitation of the CBMM, our current work is limited in its scope of application. We have only characterized the RBC aggregate disaggregation under Couette flow shearing conditions but have not presented the scenarios of blood flow in vessel lumens. In such rheological scenarios, there will be weak shear in the lumen center and very high shear rates near the vessel walls. We expect RBC aggregate disaggregation mechanisms in the near-wall regions to be similar to the OT-based shearing mechanisms. Flow pulsatility is also another aspect of physiological flows that cannot be discussed in the present study since we have only applied doublet disaggregation at one constant pulling rate (0.15 μm·s^−1^). While these two aspects are invaluable features of physiological blood flow, we feel that without experimental data the model predictions in such scenarios may be pure speculation. As such we have limited the scope of this paper to data available from present CFS experiments.

## Conclusion

In summary, we have developed a theoretical framework for describing cross-bridge dynamics in plasma-mediated RBC aggregation through our cross-bridge migration model. By combining the observations from Fg proteomic studies and RBC-doublet level force spectroscopy techniques, we have demonstrated the parametric workings of a spatially non-uniform adhesion dynamics model that prescribes a surface convection-diffusion transport of a mobile cross-bridging Fg complex. Our simulation results for doublet formation indicate that RBC-RBC adhesion mechanisms in plasma are likely weak and non-specific based on the kinematics of inter-RBC motion required for contact area growth. Our doublet disaggregation simulations show that the inverse force to contact area relationship reported by doublet disaggregation experiments can be predicted by the CBMM under the condition of an mFg diffusivity that is three orders of magnitude lower than aqueous Fg diffusivity (1.8x10^−11^ → 1 × 10^−15^ m^2^∙s^−1^). As we have not explicitly included bridge formation reaction kinetics in the CBMM, the effective diffusivity may be a qualitative representation of the diffusion-limiting effects of local cross-bridge formation and disassembly. Furthermore, the finding at least qualitatively demonstrates the important role of RBC surface interactions with mFg in mediating cross-bridge migration and inter-RBC affinity. We also demonstrated how the CBMM can be staged to qualitatively represent clinical ranges of healthy and diseased RBC aggregation and disaggregation dynamics.

## Data Availability

The original contributions presented in the study are included in the article/Supplementary Material, further inquiries can be directed to the corresponding author.
