# Supplementary material for: A mechanistic model of cross-bridge migration in RBC aggregation and disaggregation
Source: Front Bioeng Biotechnol. 2022 Dec 6;10:1049878. doi: 10.3389/fbioe.2022.1049878 (PMC9763627; doi:10.3389/fbioe.2022.1049878)
Supplement: Supplementary file 8 [file DataSheet1.docx]

Supplementary Material

***Section A: Elastic constitutions of the coarse-grained particle model***

The coarse-grained particle model (CGPM) (Pivkin and Karniadakis, 2008) represented the RBC deformation dynamics on the membrane. Membrane strain energy in the CGPM was calculated on a triangular surface mesh where constitutive expressions for areal deformation energy $U_{PM area}$ and bending energy $U_{PM bend}$ of the plasma membrane (PM), shearing energy $U_{CSK shear}$ of the viscoelastic RBC cytoskeleton (CSK) and compressive energy $U_{cytosol vol}$ of the incompressible cytosolic volume determined the internal forces of the membrane deformation:

$$U_{RBC}= U_{CSK shear}+{U_{PMbend}+U}_{PM area}++U_{cytosol vol} [A1]$$

The CSK shearing strain energy is given by a combination of the worm-like chain (WLC) model that represents the entropic elasticity of stretched spectrin tetramers and power force spring model (POW) that represents the steric properties of coiled spectrin tetramers in the CSK:

$$U_{CSK shear}=\sum_{j\in\left( 1\ldots N_{e} \right)} k_{B}T\left( \frac{l_{m}}{4p_{j}} \right)\left( \frac{3\left( \frac{l_{j}}{l_{m}} \right)^{2}-2\left( \frac{l_{j}}{l_{m}} \right)^{3}}{1-\left( \frac{l_{j}}{l_{m}} \right)} \right)+\sum_{j\in\left( 1\ldots N_{e} \right)} \frac{k_{p,j}}{l_{j}} [A2]$$

where $N_{e}$ is the total number of CSK mesh edges (representing spectrin tetramers), $l_{j}$ is the length of each edge, $l_{m}$ is the contour length, $p_{j}$ is the persistence length, $k_{B}$ is the Boltzmann constant, $T$ is the temperature and $k_{p,j}$ is the chain compression spring constant. The resting shear modulus ($E_{s0}$) under infinitesimal strain is given by (Fedosov, 2010):

$$E_{s0}=\left( \frac{\sqrt{3}k_{B}T}{4p_{j}l_{j,0}} \right)\left[ \frac{\frac{l_{j,0}}{l_{m}}}{2\left( 1-\frac{l_{j,0}}{l_{m}} \right)^{3}}-\frac{1}{4\left( 1-\frac{l_{j,0}}{l_{m}} \right)^{2}}+\frac{1}{4} \right]+\frac{3\sqrt{3}k_{p,j}}{4{l_{j,0}}^{3}} [A3]$$

where $l_{j,0}$ is the mesh edge length for the RBC at rest.

The bending strain energy in the PM is given by:

$$U_{PM bend}= \sum_{j\in\left( 1\ldots N_{e} \right)} {\frac{2}{\sqrt{3}}E}_{b}\left[ 1-cos\left( \theta_{j}-\theta_{j,0} \right) \right] [A4]$$

where $\theta_{j}$ is the angle subtended by the normals of the two adjoining triangular mesh elements along their shared mesh edge *j*. $E_{b}$ is the bending modulus, $\theta_{j,0}$ is the local spontaneous angle that a free sheet of lipid bilayer will adopt in the unstressed state.

The areal strain energy in the PM is given by the area-preserving model:

$$U_{PM area}= \frac{k_{a}\left( A_{RBC}-A_{RBC,0} \right)^{2}}{2A_{RBC,0}}+\sum_{tri\in\left( 1\ldots N_{tri} \right)} \frac{k_{d}\left( A_{tri}-A_{tri,0} \right)^{2}}{2A_{tri,0}} [A5]$$

where $N_{tri}$ is the total number of PM mesh triangular elements (representing lipid bilayer patches) in the model, $A_{tri}$ is the current area of a local triangular element, $A_{tri,0}$ is the area of the element at rest, $A_{RBC}$ is the current surface area of the PM, $A_{RBC,0}$ is the PM surface area at rest (140 [µm]^2^), $k_{a}$ is the global area compressibility coefficient and $k_{d}$ is the local compressibility coefficient. The effective area compressibility modulus of the PM-CSK is given by the relation (Fedosov, 2010):

$$K=2E_{s0}+k_{a}+k_{d} [A6]$$

The hydrodynamic energy of cytosolic compression within the RBC interior is given by:

$$U_{cytosol vol}= \frac{k_{\Omega}\left( \Omega-\Omega_{0} \right)^{2}}{2\Omega_{0}} [A7]$$

where $\Omega$ is the current interior volume of the RBC, $\Omega_{0}$ is the original interior volume of the RBC at rest (100 fL) and $k_{\Omega}$ is the volume correction penalty coefficient.

***Section B: Strain-hardening and apparent shear modulus calculation in planar membranes***

**
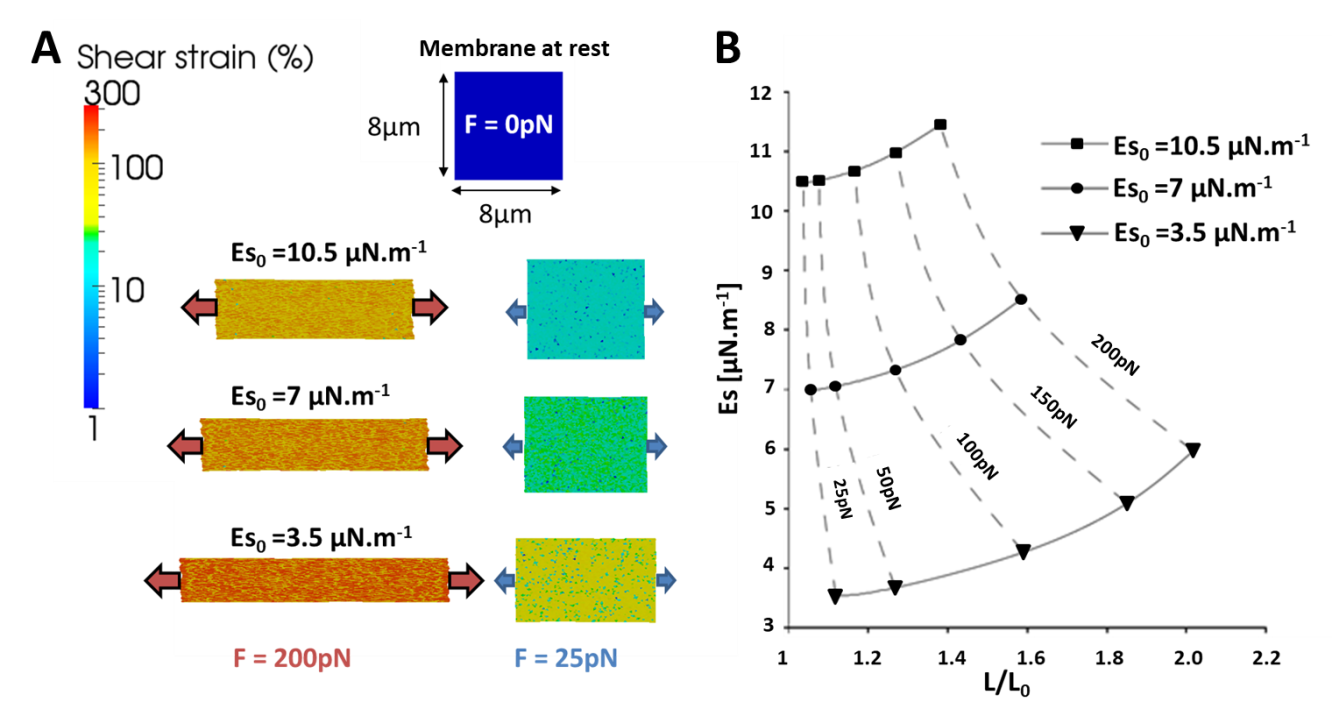
**

**Fig. S1** The coarse-grained spectrin and bilayer membrane particle model with area preservation and shear-strain hardening behavior: **A:** Shearing an 8 µm by 8 µm square membrane patch **B:** Predicted strain-hardening behavior of the RBC membrane model demonstrated on square membrane patch

With appropriate staging of $p$, $l_{m}$ and $k_{p}$ in eqn. A3 of the preceding section, the coarse-grained particle membrane model may match the experimentally observed deformations. Key to the empirical fit for RBC deformation behavior is the strain-hardening nature of the RBC membrane which we demonstrate on an 8 µm by 8 µm square membrane patch. The RBC membranes in the model are staged with the physiologically reported range of soft, normal and hard load-free shear elasticity ($E_{s0}$ = 3.5, 7 and 10.5 µN/m respectively). The apparent shear modulus ($E_{s}$) under increasing tension was calculated using the shear stress to shear strain relation for an area-conserving hyperelastic sheet (Hansen et al. 1996):

$$E_{s}=\frac{T_{s}}{\left( {\lambda_{1}}^{2}-{\lambda_{1}}^{-2} \right)/2} where T_{s}=\frac{1}{2}\left| T_{1}-T_{2} \right| [B1]$$

where $\lambda_{1}$ is the principal extension aligned with the pulling axis and may be replaced by the extension ratio $\left( \frac{L}{L_{0}} \right)$ for a flat membrane from its original length $L_{0}$. $T_{s}$ is the shear stress arising from the pulling stress $T_{1}$ which is simply the uniaxial tension $F$ divided by the width of the membrane at equilibrium deformation. $T_{2}$ is zero in the uniaxial stretching case.

Results in Fig. S1B show that the membranes strained with a shear modulus close to their resting shear moduli $E_{s0}$ at low tension ($F$ < 25 pN). However, with increasing uniaxial tension, strain-hardening behaviour of the membrane was evidenced in the model by increasing apparent shear modulus $E_{s}$.

***Section C: Validations of RBC deformability in the model against experiments in the literature***

***
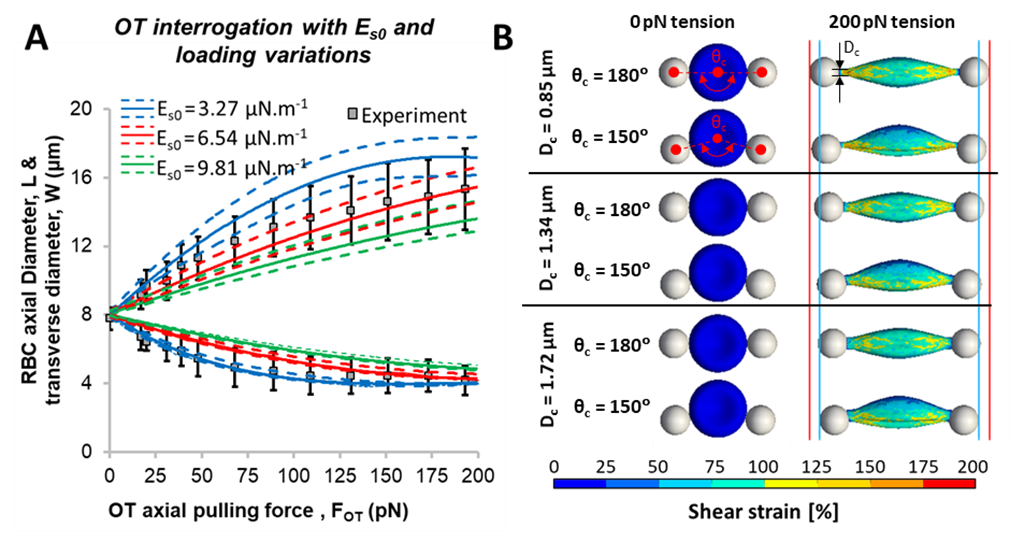
***

**Fig. S2** The RBC axial extension and transverse compression produced by a uniaxial stretching force. **A:** Results of the extension in relation to the cellular variation in resting shear elastic modulus E_s0_ and geometric inconsistencies in the loading conditions. **B:** The shear strain distribution on the cell membrane corresponding to 200 pN tension for membrane model with E_s0_ = 6.54 μN.sm^-1^ (red group in A) and under varying loading contact area and positions.

Shearing modulus

The shearing deformability of RBCs in the aggregation model was validated against the optical tweezers (OT) force-probing experiments published in the literature (Suresh et al. 2005) where the resulting diameters of the deformed RBCs against the applied OT tension were measured (Fig S2A). Variations in the bead handle position in the OT experiment were represented in the *in silico* model of RBC deformation for consideration of the bead misalignment errors (θ_c_ ≠ 180°) and bead contact diameter (D_c_) variations in experiments. Even with the bead misalignment (Fig. S2B), RBC models within the physiological shearing modulus range ($E_{s0}$= 3.27 – 9.81 µN∙m^-1^) matched OT experimental data for the force-extension curves (Fig. S2A). Further verification of the shearing modulus range was assessed by the micropipette aspiration (MA) models which matched experimental MA literature (Waugh and Evans 1979) within this range (Fig. S3A). Consequently, we employed the median $E_{s0}$ (6.54 µN∙m^-1^) for an averaged representation of shearing elasticity of the RBC membrane in our aggregation models.


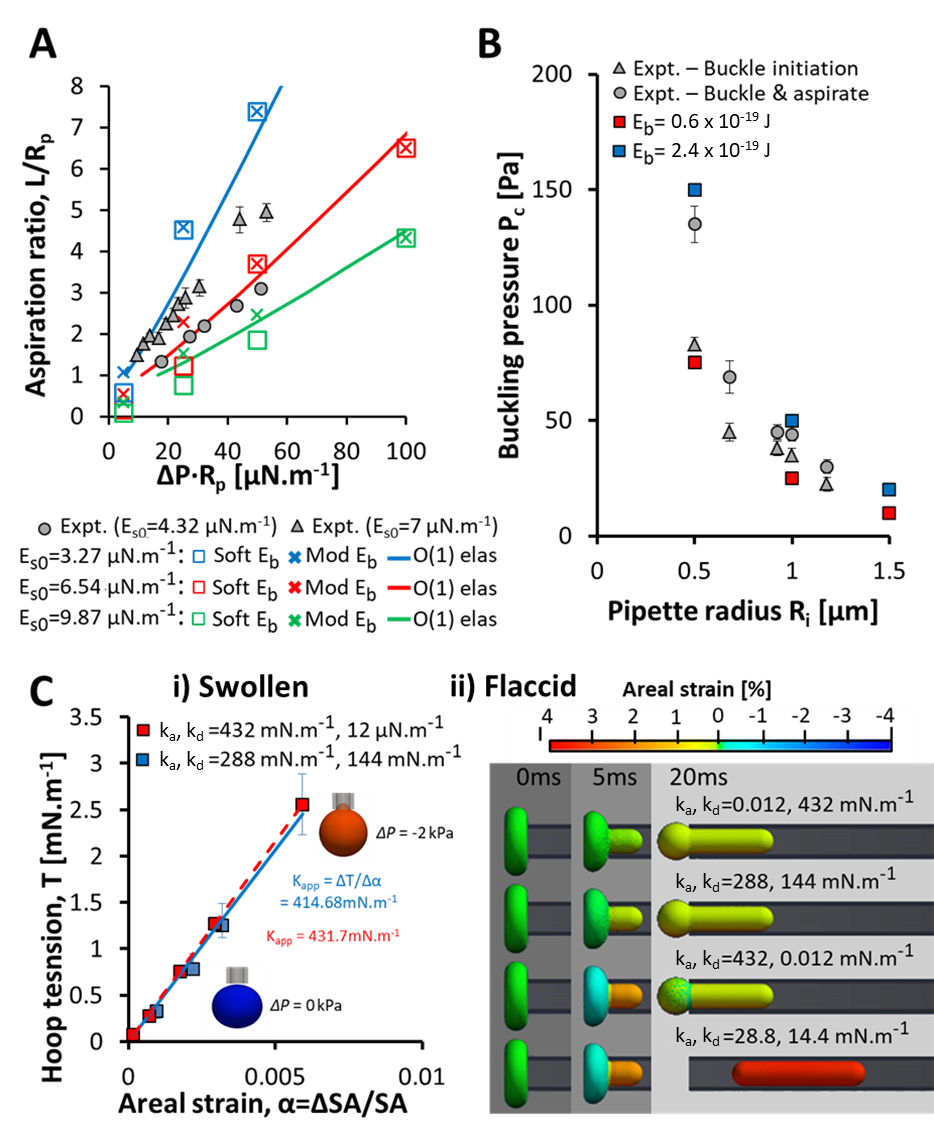


**Fig. S3** The multiple deformability modes of the RBC probed with micropipette aspiration techniques. **A** Membrane shear modulus, E_s0_ determination in small caliber pipettes utilizing the observed aspiration length, known pipette inner radius and varied suction pressure. **B** Membrane bending modulus E_b_ determination based on cell buckling limit. **Ci** Determination of membrane isotropic (areal modulus) by examining the isotropic expansion in the distended free-end of the RBC; **ii** RBCs with physiological membrane areal modulus are unable to enter completely into a 3 µm micropipette while softening of the membrane allows cell passage but at the risk of mechanical lysis due to areal strains beyond the lipid bilayer stability limits (Shigematsu et al. 2015).

Bending modulus

Next, we assessed the bending modulus range ($E_{b}$) to be employed in our RBC aggregation model by verification of the critical aspiration pressure required to buckle RBC membranes into small calibre micropipettes as reported in the experimental literature (Evans 1983). We found the appropriate $E_{b}$ range where models matched experimental aspiration mechanics to lie between (0.6 – 2.4) x10^-19^ J (Fig. S3B). Consequently, we employed the $E_{b}$ matching the buckle and aspiration initiation limit in MA experiments (2.4x10^-19^ J) for a representation of physiological flexural elasticity of the RBC membrane in our aggregation models.

Area dilation (areal) modulus

The final elasticity characteristic we assessed for the RBC deformation model was the areal modulus given by the expression in A6. In the MA test with swollen RBCs in hypotonic medium, the pre-swollen RBCs were aspirated under high pressure that produces areal expansion of the RBC thereby allowing investigators to estimate the areal modulus (Evans et al. 1976). In Fig. S3Ci we show that following the expression in A6, the variation between the local dilation modulus $k_{d}$ and global dilation modulus $k_{a}$ does not affect the distribution of hoop tension from the areal expansion in the high pressure MA model with swollen RBCs for as long as the summation of $k_{a}$ and $k_{d}$ leads to the same magnitude of $K$ (~432 mN∙m^-1^) (Evans 1989). In the case of a flaccid RBC entering into a 3µm internal-diameter microtube (Fig. S3Cii), variation in the $k_{a}$ to $k_{d}$ permutation affects the spatiotemporal distribution of areal strain in the physiological model (first 3 rows). A reduction in the overall $K$ by ten times (43.2 mN∙m^-1^) in the fourth example shows the importance of areal expansion resistance in limiting RBC deformability and cell passage in high aspiration scenarios. Consequently, we employed a value of $K$ = 432 mN∙m^-1^ in our aggregation modeling study.

RBC membrane viscosity

The reported range for RBC membrane viscoelasticity is dependent on the stress protocol applied on the cell. In the case of OMTC, the torsional deformations are characterized by the elastic storage modulus g’ and viscous loss modulus g”. We noted that elements of the OMTC experiment that could not be controlled for such as the variation in magnetic bead contact area (Fig. S4Ai) and variation in the RBC membrane bending resistance (Fig. S4Aii) can affect the g’ and g” prediction, however these variations did not alter the cross-over frequency (f*) where the g’ and g” curves intersect. Variation in membrane viscosity however, altered the dynamic balance between elastic and viscous property in the RBC membrane as f* occurred at lower frequencies in membranes with higher viscosity (Fig. S4Aiii). The cross-over frequency f* can be understood to be the frequency of deformation at which the viscous dissipation in the membrane matches the elastic energy stored in the membrane from the deformation – deformation rates occurring above f* result in highly viscous behavior of the RBC membrane where most of deformation work is dissipated and unrecoverable after elastic restoration of the membrane shape. Correspondence with the OMTC experiment (Puig-de-Morales-Marinkovic et al. 2007) is obtained for simulated models employing membrane viscosities in the lower ranges of 0.023 – 0.035 µN.s.m^-1^ (Fig. S4Aiii).

In the case of recovery of the cell shape following a large extensional deformation of the entire cell surface, the shape recovery (L/W) has been characterized by the recovery time constant $t_{c}$. Various experiments utilizing a wide variety of extensional techniques have reported $t_{c}$ in the range of 0.1 – 0.19 s (Hochmuth et al. 1979, Mills et al. 2004). For an RBC membrane with a shear modulus of 6.54 µN.m^-1^, the corresponding membrane viscosity applied in the model in order to match the experimental recovery phenomena was 0.7 µN.s.m^-1^. Despite the wide variation in initial stretching ratio ((L/W)_m_ = 1.368 – 3.889) applied in the models, the predicted values of $t_{c}$ was well within the reported experimental range (Fig. S4Bi), thus indicating that the membrane viscosity value of 0.7 µN.s.m^-1^ well represented shape recovery dynamics for large deformation scenarios. Naturally, the contradictory dichotomy of low membrane viscosity predicted by OMTC and high membrane viscosity predicted by large extensional deformation recovery experiments indicate a complex surface viscosity mechanism that remains unaccounted for in our present simulation models. We have assumed that the shearing motions in our aggregation and disaggregation scenarios match the stress protocols in the large extensional deformation scenario and have employed a membrane viscosity of 0.7 µN.s.m^-1^ in our aggregation modelling study.


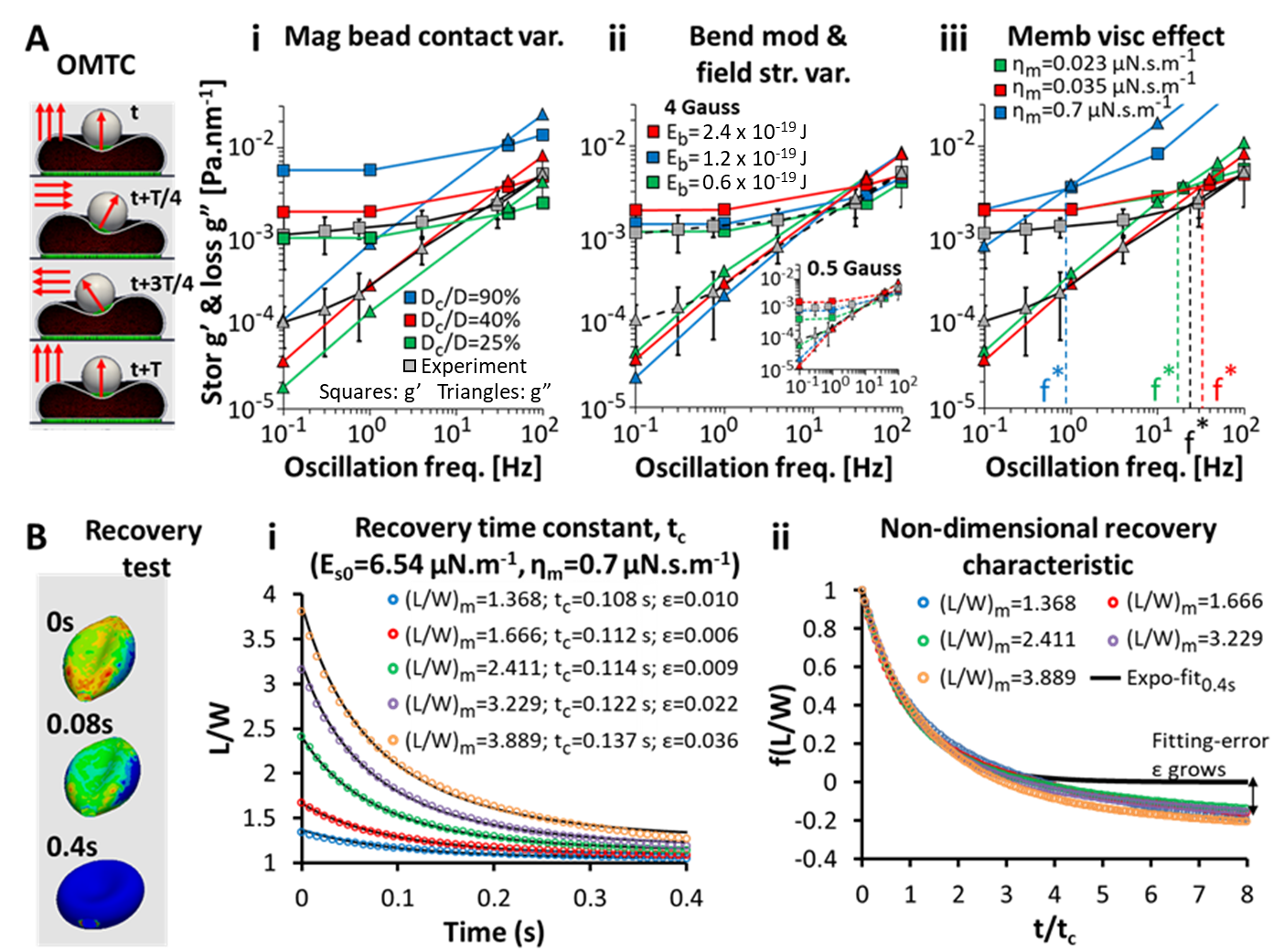


**Fig. S4** RBC viscoelasticity characterization with the OMTC test (**A**) and uniaxial recovery test (**B**).

***Section D: Secondary validation of RBC surface affinity through parametric optimization of the depletion model for RBC aggregation against spherical encapsulation experiment***

Depletion theory interaction model of RBC aggregation

In a mixed suspension of polymer solutes and larger insoluble colloid particles, a depletion layer forms around each colloid particle when conformational entropy restrictions on polymers are not compensated by their adsorption onto the colloid surface (Asakura and Oosawa 1954). When colloid surfaces are separated by distances smaller than their combined depletion layer, the resulting osmotic imbalance brings colloid particles together to almost contact. Similarly, RBCs suspended in buffer solution with polymers such as dextran experience depletion-mediated attraction in accordance with the depletion layer thickness unique to the polymer type and concentration. Since the RBC has a negative surface charge, contact between cells is prevented by the short-ranged electrostatic repulsion within the electric double layer between cells. Consequently, the total RBC-RBC interaction ($\Gamma_{total}$) in polymer suspension is given by a superposition of the depletion interaction energy ($\Gamma_{dep}$) and the electrostatic repulsion energy ($\Gamma_{e}$) (Neu and Meiselman 2002):

$$\Gamma_{total}=\Gamma_{dep}+\Gamma_{e} [D1]$$

The depletion interaction energy is given by:

$$\Gamma_{dep}=\left\{ \begin{aligned} P_{osmo}\left( 2\left( \Delta+\delta-p \right)-r_{sep} \right) r_{sep}\leq2\left( \Delta+\delta-p \right) \\ 0 r_{sep}>2\left( \Delta+\delta-p \right) \end{aligned} \right. [D2]$$

where $P_{osmo}$ is the osmotic pressure, $\Delta$ is the depletion layer thickness for smooth hard surfaces, $\delta$ is the glycocalyx height on the RBC surface, $p$ is the penetration depth of free polymers into the glycocalyx and $r_{sep}$ is the inter-membrane separation distance above the glycocalyx layers. Polymer solute theory provides the $P_{osmo}$ from the virial expansion of solute-solvent particle interaction:

$$P_{osmo}=\frac{RT}{M}c_{b}+B_{2}c_{b}^{2} [D3]$$

where $R$ is the universal gas constant, $T$ is the temperature, $M$ and $c_{b}$ are the molecular weight and bulk concentration of the flocculation-inducing macromolecule and $B_{2}$ is the second virial coefficient whose value for dextrans is given in (Haynes et al. 1989, Ioan et al. 2001, Nordmeier 1993).

The depletion layer thickness for a hard smooth surface is given as follows:

$$\Delta=-\frac{1}{2}\frac{P_{osmo}}{\chi}+\frac{1}{2}\sqrt{\left( \frac{P_{osmo}}{\chi} \right)^{2}+4{\Delta_{0}}^{2}} where \chi=\frac{2k_{B}T}{{\Delta_{0}}^{2}}\left( \frac{c_{b}N_{a}}{M} \right)^{\frac{2}{3}} [D4]$$

where $\Delta_{0}$ is the maximum depletion layer thickness given to be 1.4 times of the radius of gyration of the bulk polymer (Vincent et al. 1986).

Bulk polymer penetration depth into the glycocalyx $(p)$ is given as follows:

$$p=\delta\left( 1-e^{-c_{b}/c_{p}} \right) [D5]$$

where $c_{p}$ is the penetration constant signifying the bulk polymer concentration at which 63% penetration of the glycocalyx occurs ($c_{b}=c_{p}$).

The electrostatic repulsion energy is given by:

$$\Gamma_{e}=\frac{\sigma^{2}}{\delta^{2}\varepsilon\varepsilon_{0}\kappa^{3}}\left\{ \begin{aligned} \sinh\left( \kappa\delta\right)\left( e^{\kappa\delta-\kappa r_{sep}}-e^{-\kappa r_{sep}} \right) r_{sep}\geq2\delta\\ \left( 2\kappa\delta-\kappa r_{sep} \right)-\left( e^{-\kappa\delta}+1 \right)\sinh\left( \kappa\delta-\kappa r_{sep} \right)-\sinh\left( \kappa\delta\right)e^{-\kappa r_{sep}} r_{sep}<2\delta\end{aligned} \right. [D6]$$

where $\sigma$ is the surface charge density on the RBC surface due to sialic residues in the glycocalyx, $\kappa^{-1}$ is the Debye-Huckel length and $\varepsilon\varepsilon_{0}$ is the electrical permittivity of the solute.

When the surface separation distance $r_{sep}$ is larger than the effective double-depletion-layer width ($\Delta_{eff}=2\left( \Delta+\delta-p \right)$) flocculation does not occur; note the contribution of the glycocalyx height in increasing $\Delta_{eff}$ whereas polymer penetration into the glycocalyx decreases $\Delta_{eff}$. When $r_{sep}$ is smaller than $\Delta_{eff}$, depletion attraction causes two mating surfaces to flocculate. When the separation distance approaches the double glycocalyx layer width ($2\delta$) electrostatic repulsion produces strong repulsion forces preventing surface to surface contact. Using Eq. (D1), (D2) and (D5), the net interaction energy $\Gamma_{total}$ against $r_{sep}$ can be predicted as shown in Fig. S5C. The adhesivity or affinity of the net attraction at any given $c_{b}$ is indicated by the minimum point (energy-minimization state) on the $\Gamma_{total}$-$r_{sep}$ curve in the depletion-electrostatic superposition.

The depletion interaction model for RBCs proposed by (Neu and Meiselman 2002) has been successfully employed to study the effects of cellular factors on the RBC aggregation, which include alterations in the height and charge density of the glycocalyx (Rad et al. 2014), RBC shape variation from solution tonicity changes and RBC aging (Neu et al. 2003).

Least-square error optimization of depletion model parameters and surface affinity determination using encapsulation test

The encapsulation of a spherical RBC fragment by a flaccid RBC is the classic experiment (Buxbaum, Evans and Brooks 1982) following JKR theory for calculating surface affinity in deformable adhesive interfaces (Johnson et al. 1971). The simple basis for this test is to relate RBC deformation to surface affinity energy by understanding the new equilibrium of the two-body interaction as a balance between the gain in elastic energy of the surface deformation and the reduction in surface energy from the adhesive interaction. Using the encapsulation to RBC affinity curve obtained from the numerical simulation we estimated the RBC affinity to dextran concentration by matching the experimental encapsulation data with our numerical model prediction – this was done by identifying the affinity value at which the experimental encapsulation ratio ($\frac{Z_{e}}{2R}$) intercepts with the encapsulation-affinity numerical curve (black and red curve) in Fig. S5A. Figures S5B & C demonstrate the parametric workings of polymer concentration, penetration on depletion width and osmotic potential. Figure S5D shows our estimation of the 70kDa and 150kDa dextran (D70 and D150) depletion-mediated RBC-RBC affinity to dextran concentration using a least-square curve-fitting of the depletion model optimized for parameters in C2, C4 – C6 annotated in red: we estimate the D70-mediated affinity to peak at 4.17µJ.m^-2^ for 3g/dL of D70 in PBS solution while the D150-mediated affinity to peak at 9.03µJ.m^-2^ for 4.8g/dL of D150 in PBS solution.

**
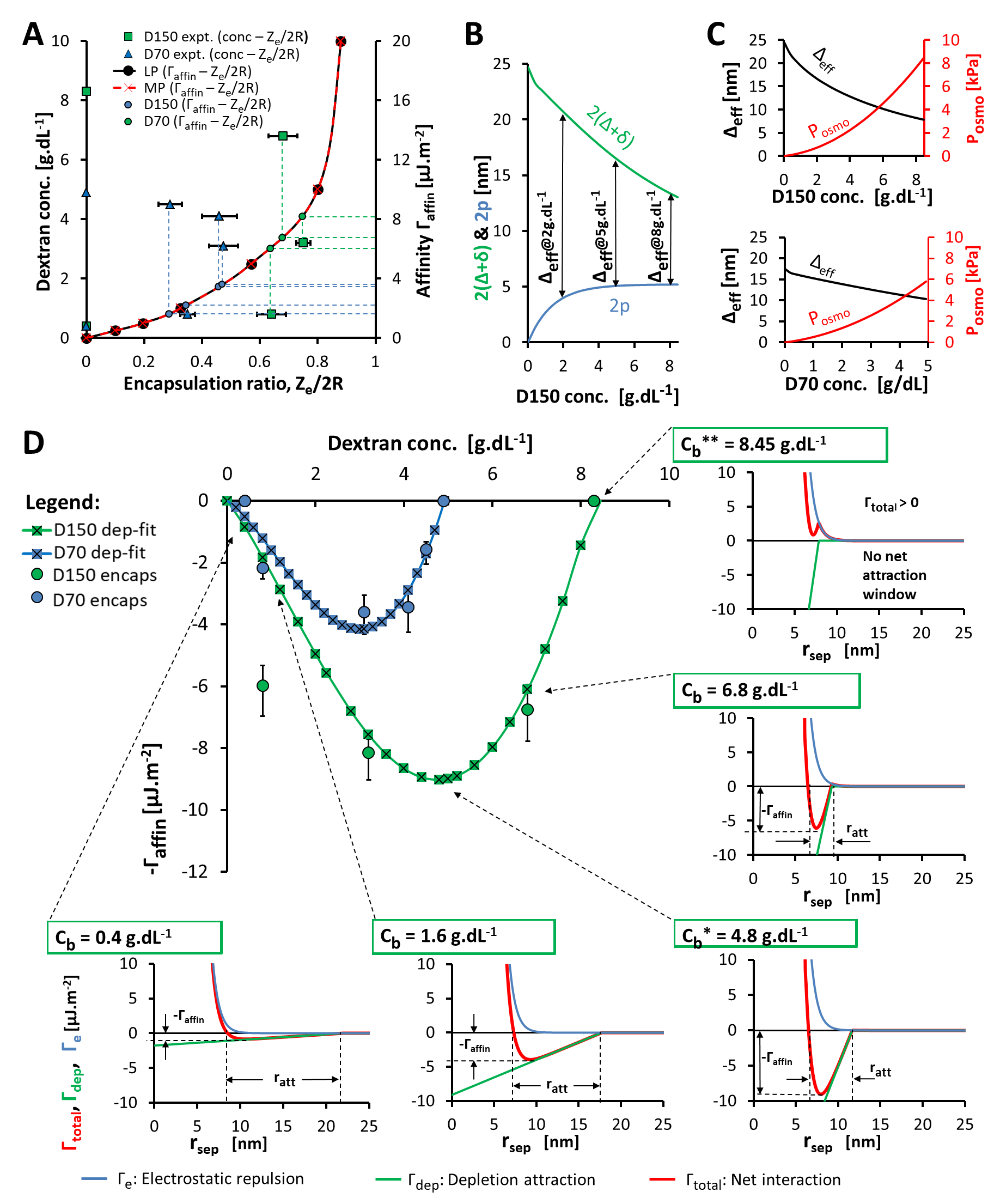
**

**Fig. S5:** Dextran concentration, encapsulation ratio and adhesion affinity phase diagram **(A)** used to determine the dextran concentration to adhesion affinity envelopes obtained from fitting the depletion theory model (Neu and Meiselman 2002) (with parametric optimization) to empirical results in **(D)**. Figure (**B**) shows the reduction of the effective double depletion layer due to an increasing penetration effect (eqn. D5) and increasing osmotic pressure (eqn. D4) while figure (**C**) highlights the effect of an increasing bulk polymer concentration on increasing the pressure of attraction (P_osmo_; eqn. D3) between surfaces while being mitigated by the reducing depletion layer width (eqn D4) – these two mechanisms results in the bell-shaped affinity-to-concentration for depletion-induced adhesion in (**D**)


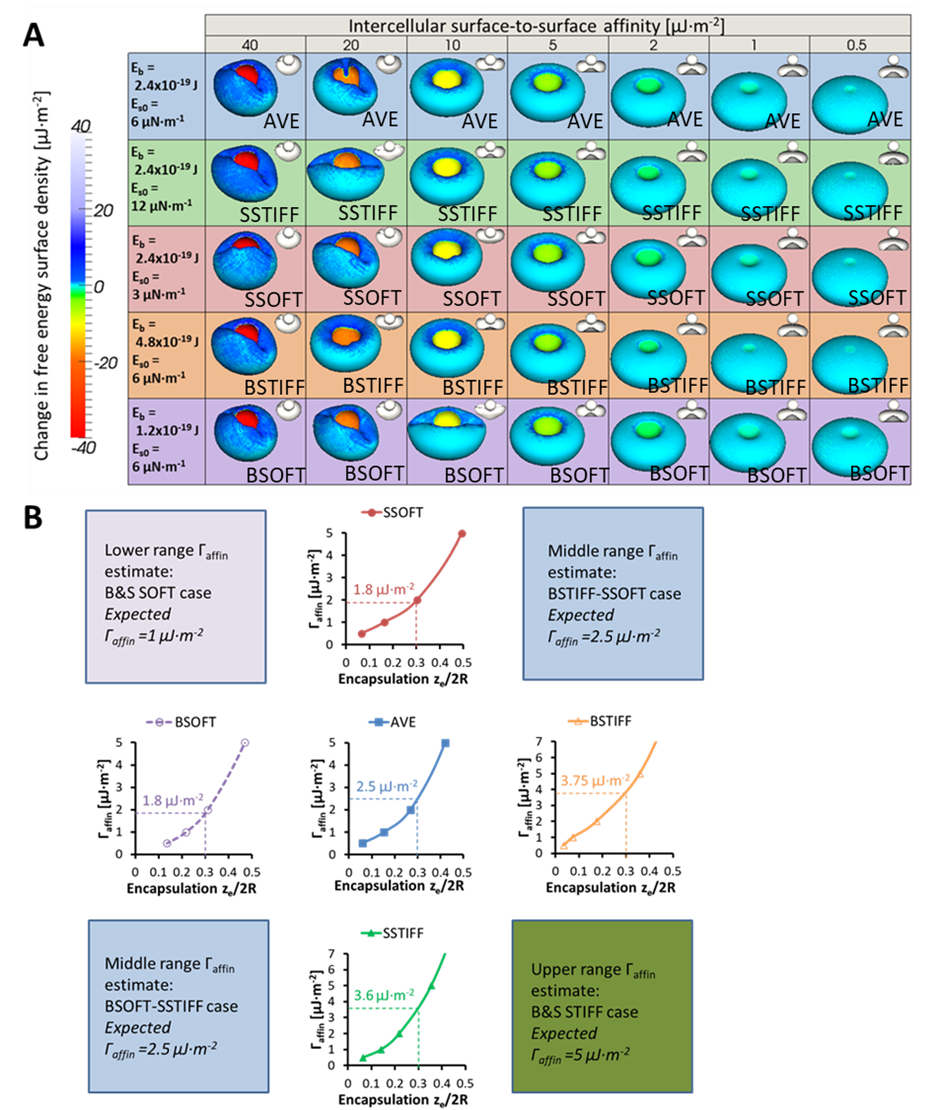


**Fig. S6:** **A**: Encapsulation under varying membrane deformability scenarios.

**B**: the corresponding range of predicted surface affinities dictating inter-RBC attraction in plasma based on the encapsulation ratio of 0.3 (Buxbaum et al. 1982).

**See simulation movies in SM8 & SM9.**

After establishing the theoretical envelopes of the encapsulation ratio to surface affinity, we could revisit the seminal work of (Buxbaum, Evans and Brooks 1982) where the encapsulation ratio of spherical fragments by flaccid RBCs was reported to be ~0.3.

Based on the physiological variation in RBC membrane deformability, the surface affinity for RBC-RBC attraction in blood plasma was found by the encapsulation analysis to operate in a wide range from 1 to 5µJ∙m^-2^. This wide variation arises due to the wide range in RBC deformability and the fundamental principle of the encapsulation analysis that links deformation energy to surface affinity in the energy minimization state of the doublet interaction. Hence in our main text we calibrated the average surface affinity value using a different approach. In the adopted approach of surface affinity evaluation, we employed the doublet formation kinematics test instead, which we do not expect to be as sensitive to RBC membrane deformability variations as the encapsulation analysis.

***Section E: Physio-chemistry of fibrinogen, the role of sub-domains promoting RBC aggregation***


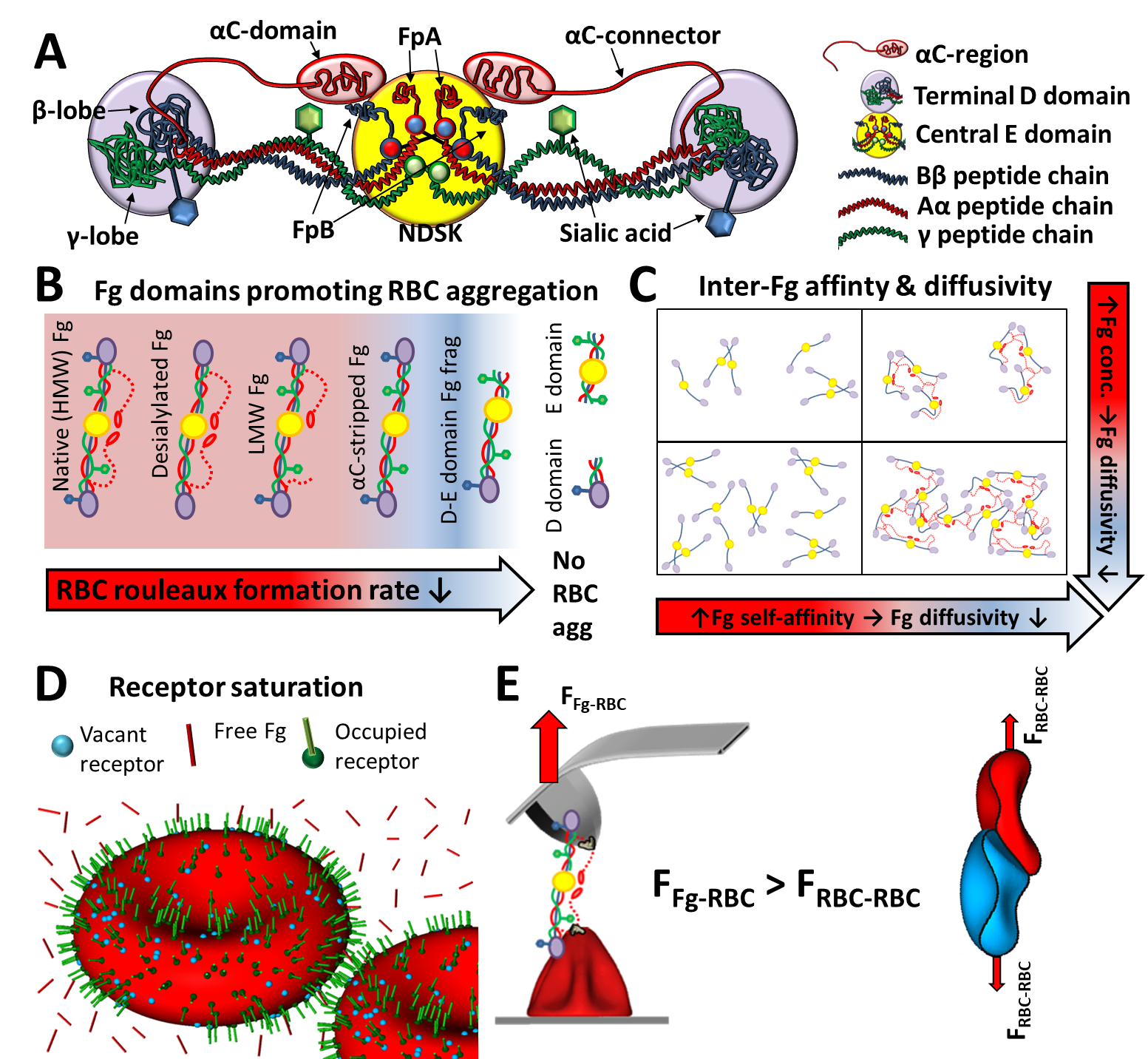


**Fig. S7** The known structure of fibrinogen Fg (**A**), its domain involvement in promoting RBC aggregation (B), its attenuated diffusivity due to non-specific Fg-Fg interactions (**C**), an almost saturated specific attachment of Fg (ligand) to available integrin-associated receptors on the RBC surface under physiological systemic Fg levels (**D**), the observation that Fg-RBC receptor rupture force is higher than the RBC-doublet fracture force and our proposed theoretical framework (inset) for the evaluation of the implied mechanisms from these earlier studies **(E)**.

A key protein participating in the mechanism of plasma-mediated RBC aggregation is fibrinogen (Fg), a ~340 kDa protein that consists of a dimer arrangement of two tri-chain (Aα, Bβ and γ) polypeptides joined at the Fg central E domain with the N-terminal disulphide knot (NDSK). At its two C-terminals, Bβ and γ form their respective lobes that make up Fg’s distal D domains while αC extends from the tri-chain backbone to interact with its counterpart αC (from the pairing monomer) and the peptide groups FpA and FpB in the E domain (Zuev et al. 2017). On the role of Fg in RBC aggregation, several key observations have been made:

1) Specificity of Fg’s interaction with an integrin-associated receptor on the RBC has been well documented through proteomic interrogation (De Oliveira et al. 2012, Lominadze and Dean 2002, Sokolova et al. 2014). Force spectroscopy studies with atomic force microscopes (AFM) have reported binding-rupture events between Fg and RBC surface receptors occurring with rupture forces in the range of 20 – 80 pN (Carvalho et al. 2010, Carvalho et al. 2011);

2) the observed differences in RBC rouleaux formation speed with Fg-variants and Fg-fragments has highlighted the importance of the αC region in promoting spontaneous RBC aggregation (Maeda et al. 1987) (Fig. S6B);

3) the diffusivity of Fg in solution decreases with increasing Fg concentration possibly due to Fg nucleation through αC entanglement between Fg (Zuev, Litvinov, Sitnitsky, Idiyatullin, Bakirova, Galanakis, Zhmurov, Barsegov and Weisel 2017) (Fig. S6C);

4) the specific-binding of Fg to the RBC surface has been estimated to be almost saturated at physiological concentrations of Fg (Lominadze and Dean 2002) (Fig. S6D);

5) the RBC-doublet fracture force (~30pN (Khokhlova et al. 2012, Lee et al. 2016)) is lower than the rupture force measured between single Fg to RBC specific binding (20 – 80 pN (Carvalho, Connell, Miltenberger-Miltenyi, Pereira, Tavares, Ariens and Santos 2010, Carvalho, de Oliveira, Freitas, Gonçalves and Santos 2011)) (Fig. S6E);

6) RBC doublets resuspended in Fg-albumin (Alb) solution exhibit similar force dissociation thresholds as RBC doublets in plasma but spontaneous doublet formation from two point-contacted cells only occurs in plasma (Lee, Kinnunen, Khokhlova, Lyubin, Priezzhev, Meglinski and Fedyanin 2016).

Observations 1 – 3 demonstrate the capacity of Fg for both specific and non-specific Fg-Fg and Fg-RBC interactions which may be mediated by the physiochemistry of Fg structure and mobility while 4 suggests that the scenario of a single Fg bridging receptors from two neighboring RBC surfaces is unlikely given that vacant receptor sites are rare under physiological bulk Fg concentration. Observation 5 when considered with 1 – 4, suggests that the bridge between two adhering RBCs is more likely non-specific in that 1) free domains of an Fg specifically bound to one RBC either adsorbs to the surface of the pairing RBC non-specifically or 2) interacts with a pairing Fg bound to the neighboring RBC through non-specific interaction between their free domains or 3) through the mediation of a third (or more) Fg non-specifically entangled with the two bound Fg. In all three scenarios, bridge rupture will be dictated thus, by the weakness of the non-specific interactions and not by the strength of the specific-binding between Fg and the integrin-associated receptor on the RBC. Finally, observation 6 highlights the possibility of synergistic cooperation between plasma proteins in aggregate formation while suggesting Fg and Alb to be dominant factors for aggregate stability in the aggregate dispersion process.

***E Simulation movies***

***SM1:*** The multiple bridging modes considered: Cross-bridging in group A contained only mobile non-specific cross-bridges (mFgB) and employed VTS adhesion. Group B consisted of only non-specific immobile cross-bridging (iFgB) and employed VTS adhesion. Group C represented a mixed cross-bridge scenario containing both mobile non-specific cross-bridges (mFgB) employing VTS and immobile specific cross-bridges (iFgRB) employing VTV. Group D represented another mixed cross-bridge scenario but with only immobile cross-bridges. The first fraction was the non-specific immobile cross-bridges (iFgB) prescribing VTS adhesion. The second fraction was specific cross-bridges (iFgRB) prescribing VTV adhesion. Group E consisted solely of specific cross-bridges (iFgRB) following VTV adhesion. A full description of simulation parameters defining the 15 sets of simulations can be found in Table 2

***SM2:*** Sliding formation of RBC doublet aggregates with vertex to surface (VTS; top row) and vertex to vertex (VTV; bottom row) adhesion schemes. Sliding and friction dynamics produce mobile cross-bridge (mFg) migration as prescribed by the cross-bridge migration model (CBMM) in the VTS representation of non-specific RBC surface interactions. On the other hand the VTV which represents specific interactions is assumed to be physically incompatible with the concept of migrating bridging sites or tethers and hence is a uniform affinity model (UAM). Furthermore, the VTV adhesion is limited by adhesion vertex availability and short cross-bridging-length. Consequently, VTS models slide to spontaneously increase adhesion contact area but the VTV models do not.

***SM3:*** Three models comparing frictional and length-scaling parameters. A reduction in friction increases formation (and inter-RBC sliding) speed. Coarse-graining of the interaction length-scale with the long-range MP model led to unnaturally fast aggregate formation.

***SM4:*** Sliding dissociation dynamics simulations of RBC doublets employing the UAM. Displacement of the diametrically opposite ends of the two RBCs in the doublet at 0.15µm∙s^-1^ produced eventual fracture of the doublet. In the process, trap tensions to pull at the constant displacement rate were obtained and analyzed against measurements published by optical tweezers studies. An increase in adhesion affinity lead to a larger adhesion contact area for the same level of doublet extension between models and also required a higher deformation level prior to fracture.

***SM5:*** Simulations of the CBMM mediated mFg accumulation in three doublet dissociation models. The top left model has a high diffusivity (D_mFg_) that does not allow mFg cross-bridges to accumulate in the intercellular space. The top right model with reduced mFg diffusivity permits accumulation of mFg via frictional drag at the contact rims of the adhesion space. The bottom right model employs the same CBMM parameters as the top right model but considers out-of-surface diffusion flux – resulting in a replenishing of the depleted regions of mFg but also a diffusive loss of mFg in the adhesion zone especially during the final point-adhesion stage of the doublet dissociation (around time: 32 s in the movie)

***SM6:*** An expansive demonstration of the normal diffusion flux effect on depleted regions and adhesion zones.

***SM7:*** A parametric variation in relative surface concentration (N*) and relative affinity (Γ*)referenced against the lower range of OT dissociation force-displacement curve for healthy RBCs (where N* and Γ* are taken to be 1) in plasma can explain the clinical range of Fg and the experimental range in disaggregation forces. Likewise, elevated disaggregation forces in systemic lupus erythematosus can be explained by increases in the N* and Γ* levels.

***SM8:*** Free-surface energy minimization states predicted in spherical encapsulation models for flaccid RBCs with physiological variation in membrane elastic proprieties and variations in surface affinity.

***SM9:*** The shear strain and bending strain distribution maps of the RBC deformations under surface adhesion with varying affinity and membrane elastic properties in the spherical encapsulation modeling (refer to figure S6A for definitions of the elasticity parameters in each model).

**REFERENCES**

Asakura S, Oosawa F. 1954. On Interaction between Two Bodies Immersed in a Solution of Macromolecules. The Journal of chemical physics.22:1255-1256.

Buxbaum K, Evans E, Brooks DE. 1982. Quantitation of surface affinities of red blood cells in dextran solutions and plasma. Biochemistry. Jun;21:3235-3239.

Carvalho FA, Connell S, Miltenberger-Miltenyi G, Pereira SV, Tavares A, Ariens RA, Santos NC. 2010. Atomic force microscopy-based molecular recognition of a fibrinogen receptor on human erythrocytes. ACS nano. Aug 24;4:4609-4620.

Carvalho FA, de Oliveira S, Freitas T, Gonçalves S, Santos NC. 2011. Variations on Fibrinogen-Erythrocyte Interactions during Cell Aging. Plos One.6:e18167.

De Oliveira S, de Almeida VV, Calado A, Rosário HS, Saldanha C. 2012. Integrin-associated protein (CD47) is a putative mediator for soluble fibrinogen interaction with human red blood cells membrane. Biochimica et Biophysica Acta (BBA) - Biomembranes. 2012/03/01/;1818:481-490.

Evans EA. 1983. Bending elastic modulus of red blood cell membrane derived from buckling instability in micropipet aspiration tests. Biophysical journal.43:27-30.

Evans EA. 1989. [1] Structure and deformation properties of red blood cells: Concepts and quantitative methods. In: Methods in Enzymology. Academic Press. p. 3-35.

Evans EA, Waugh R, Melnik L. 1976. Elastic area compressibility modulus of red cell membrane. Biophysical journal.16:585-595.

Fedosov, D.A. (2010). *Multiscale modeling of blood flow and soft matter.* Doctor of Philosophy PhD, Brown University.

Hansen JC, Skalak R, Chien S, Hoger A. 1996. An elastic network model based on the structure of the red blood cell membrane skeleton. Biophysical journal. Jan;70:146-166.

Haynes CA, Beynon RA, King RS, Blanch HW, Prausnitz JM. 1989. Thermodynamic properties of aqueous polymer solutions: poly(ethylene glycol)/dextran. The Journal of Physical Chemistry. 1989/07/01;93:5612-5617.

Hochmuth RM, Worthy PR, Evans EA. 1979. Red cell extensional recovery and the determination of membrane viscosity. Biophysical journal.26:101-114.

Ioan CE, Aberle T, Burchard W. 2001. Light Scattering and Viscosity Behavior of Dextran in Semidilute Solution. Macromolecules. 2001/01/01;34:326-336.

Johnson KL, Kendall K, Roberts AD. 1971. Surface Energy and the Contact of Elastic Solids. Proceedings of the Royal Society of London Series A, Mathematical and Physical Sciences.324:301-313.

Khokhlova MD, Lyubin EV, Zhdanov AG, Rykova SY, Sokolova IA, Fedyanin AA. 2012. Normal and system lupus erythematosus red blood cell interactions studied by double trap optical tweezers: direct measurements of aggregation forces. Journal of biomedical optics. Feb;17:025001.

Lee K, Kinnunen M, Khokhlova MD, Lyubin EV, Priezzhev AV, Meglinski I, Fedyanin AA. 2016. Optical tweezers study of red blood cell aggregation and disaggregation in plasma and protein solutions. Journal of biomedical optics.21:035001-035001.

Lominadze D, Dean WL. 2002. Involvement of fibrinogen specific binding in erythrocyte aggregation. Febs Lett. Apr 24;517:41-44.

Maeda N, Seike M, Kume S, Takaku T, Shiga T. 1987. Fibrinogen-induced erythrocyte aggregation: erythrocyte-binding site in the fibrinogen molecule. Biochimica et biophysica acta. Nov 2;904:81-91.

Mills JP, Qie L, Dao M, Lim CT, Suresh S. 2004. Nonlinear Elastic and Viscoelastic Deformation of the Human Red Blood Cell with Optical Tweezers. Molecular \& Cellular Biomechanics.1.

Neu B, Meiselman HJ. 2002. Depletion-mediated red blood cell aggregation in polymer solutions. Biophysical journal.83:2482-2490.

Neu B, Sowemimo-Coker SO, Meiselman HJ. 2003. Cell-cell affinity of senescent human erythrocytes. Biophysical journal. Jul;85:75-84.

Nordmeier E. 1993. Static and dynamic light-scattering solution behavior of pullulan and dextran in comparison. The Journal of Physical Chemistry. 1993/05/01;97:5770-5785.

Pivkin, I.V., and Karniadakis, G.E. (2008). Accurate coarse-grained modeling of red blood cells. *Phys Rev Lett* 101(11)**,** 118105.

Puig-de-Morales-Marinkovic M, Turner KT, Butler JP, Fredberg JJ, Suresh S. 2007. Viscoelasticity of the human red blood cell. American Journal of Physiology-Cell Physiology. 2007/08/01;293:C597-C605.

Rad S, Meiselman HJ, Neu B. 2014. Impact of glycocalyx structure on red cell-red cell affinity in polymer suspensions. Colloids and surfaces B, Biointerfaces. Nov 1;123:106-113.

Shigematsu T, Koshiyama K, Wada S. 2015. Effects of Stretching Speed on Mechanical Rupture of Phospholipid/Cholesterol Bilayers: Molecular Dynamics Simulation. Sci Rep. Oct 16;5:15369.

Sokolova IA, Muravyov AV, Khokhlova MD, Rikova SY, Lyubin EV, Gafarova MA, Skryabina MN, Fedyanin AA, Kryukova DV, Shahnazarov AA. 2014. An effect of glycoprotein IIb/IIIa inhibitors on the kinetics of red blood cells aggregation. Clinical hemorheology and microcirculation.57:291-302.

Suresh S, Spatz J, Mills JP, Micoulet A, Dao M, Lim CT, Beil M, Seufferlein T. 2005. Connections between single-cell biomechanics and human disease states: gastrointestinal cancer and malaria. Acta Biomaterialia. 2005/01/01/;1:15-30.

Vincent B, Edwards J, Emmett S, Jones A. 1986. Depletion flocculation in dispersions of sterically-stabilised particles (“soft spheres”). Colloids and Surfaces. 6//;18:261-281.

Waugh R, Evans EA. 1979. Thermoelasticity of red blood cell membrane. Biophysical journal.26:115-131.

Zuev YF, Litvinov RI, Sitnitsky AE, Idiyatullin BZ, Bakirova DR, Galanakis DK, Zhmurov A, Barsegov V, Weisel JW. 2017. Conformational Flexibility and Self-Association of Fibrinogen in Concentrated Solutions. The Journal of Physical Chemistry B. 2017/08/24;121:7833-7843.
